# In silico trial of baroreflex activation therapy for the treatment of obesity-induced hypertension

**DOI:** 10.1371/journal.pone.0259917

**Published:** 2021-11-18

**Authors:** John S. Clemmer, W. Andrew Pruett, Robert L. Hester

**Affiliations:** 1 Department of Physiology and Biophysics, Center for Computational Medicine, University of Mississippi Medical Center, Jackson, MS, United States of America; 2 Department of Data Sciences, John D. Bower School of Population Health, University of Mississippi Medical Center, Jackson, MS, United States of America; Scuola Superiore Sant’Anna, ITALY

## Abstract

Clinical trials evaluating the efficacy of chronic electrical stimulation of the carotid baroreflex for the treatment of hypertension (HTN) are ongoing. However, the mechanisms by which this device lowers blood pressure (BP) are unclear, and it is uncertain which patients are most likely to receive clinical benefit. Mathematical modeling provides the ability to analyze complicated interrelated effects across multiple physiological systems. Our current model HumMod is a large physiological simulator that has been used previously to investigate mechanisms responsible for BP lowering during baroreflex activation therapy (BAT). First, we used HumMod to create a virtual population in which model parameters (n = 335) were randomly varied, resulting in unique models (n = 6092) that we define as a *virtual population*. This population was calibrated using data from hypertensive obese dogs (n = 6) subjected to BAT. The resultant calibrated virtual population (n = 60) was based on tuning model parameters to match the experimental population in 3 key variables: BP, glomerular filtration rate, and plasma renin activity, both before and after BAT. In the calibrated population, responses of these 3 key variables to chronic BAT were statistically similar to experimental findings. Moreover, blocking suppression of renal sympathetic nerve activity (RSNA) and/or increased secretion of atrial natriuretic peptide (ANP) during BAT markedly blunted the antihypertensive response in the virtual population. These data suggest that in obesity-mediated HTN, RSNA and ANP responses are key factors that contribute to BP lowering during BAT. This modeling approach may be of value in predicting BAT responses in future clinical studies.

## Introduction

More than a billion people have hypertension (HTN), which is currently the world’s leading preventable risk factor for cardiovascular morbidity and all-cause mortality [1]. Even with pharmacological treatment, many patients still have uncontrolled blood pressure (BP) and are at increased risk of cardiovascular morbidity and mortality [2]. Most of these patients have primary HTN, which is associated with increased sympathetic nerve activity (SNA) [3, 4].

The neural feedback mechanism the baroreflex has a well-established role in the acute regulation of SNA and BP, but its long-term importance is likely limited due to resetting [5, 6]. However, over the past two decades, devices that electrically stimulate the carotid baroreflex have been shown to chronically suppress the sympathetic nervous system (SNS) and achieve impressive reductions in BP [7, 8]. In a randomized controlled Phase III clinical trial, the well-studied Rheos device (CVRx, Minneapolis) was used to stimulate the carotid baroreflex and substantially reduced BP in patients with uncontrolled hypertension [8, 9]. However, while cleared for use in Europe, neither this device nor the second generation Barostim Neo system has been approved by the Food and Drug Administration for HTN therapy in the US.

Because the BP response to baroreflex activation therapy (BAT) in patients is variable, it is unfortunate that clinical studies have primarily focused only on antihypertensive responses. In contrast, carefully conducted studies in canines have provided mechanistic insight into which patients are most likely to respond to BAT [8]. On one hand, the findings from these studies strongly suggest that BAT lowers BP, at least in part, through the suppression of renal sympathetic nerve activity (RSNA) [8]. On the other hand, studies in both canines and humans show that BAT chronically lowers BP even after renal denervation (7, 11, 12). Thus, these studies suggest alternative mechanisms for the BP lowering effects of BAT. In this regard, experimental findings in dogs suggest increased secretion of atrial natriuretic peptide (ANP) as another potential mediator of BAT-induced reductions in chronic BP [10, 11]. However, this possibility has never been critically tested in HTN, either experimentally or in a mathematical model.

Physiological modeling provides the ability to analyze complicated interrelated effects across multiple systems. The large mathematical model, HumMod, is comprised of over 10,000 variables and parameters and includes key physiological systems that play an integral role in BP regulation (including neural, endocrine, circulatory, and renal). HumMod has been extensively used for hypothesis generation and for understanding underlying physiological mechanisms that are not easily tested in whole animal or human experiments [11–17]. In particular, this model has been used to simulate different mechanisms of HTN [17] and for understanding the cardiovascular effects of BAT [11, 18]. More recently, we have created tools that generate virtual populations [19–21]. To our knowledge, calibrating a large physiological population model using experimental data has never been done. In the current study, we use cardiovascular renal, and hormonal data from hypertensive obese dogs subjected to BAT to calibrate a virtual population to investigate the relative contributions of RSNA and ANP to BP lowering during BAT.

## Methods

### Experimental data

Previous studies by Lohmeier et al. determined chronic responses to BAT in obese dogs using the CVRx Rheos device [22, 23]. In the most recent study reported in 2012, obese dogs at baseline were associated with increased body weight, BP, heart rate, glomerular filtration rate (GFR), and activation of the renin angiotensin system (RAS), similar to findings in humans [24]. After established HTN, electrical activation of the carotid baroreflex was sustained for 1 week [22]. The specific data from this study used for model calibration included mean arterial pressure (MAP), GFR, and plasma renin activity (PRA), both before and after BAT. The original GFR measurements (99 ± 9 ml/min baseline) in the obese dogs reflected hyperfiltration as compared to baseline GFR before obesity (72 ± 6 ml/min) [22]. Assuming a normal human GFR of 120 mL/min, all canine GFR measurements were rescaled to reflect equivalent human GFR by increasing animal values 67%.

### Physiological model

All simulations were performed using HumMod, a large model of human physiology composed of mathematical relationships derived from experimental and clinical data. This model accurately reproduces the acute and chronic physiological responses and the characteristics of many pathophysiological states [11, 17, 25]. The model, its code, and mathematical derivations are available for academic download as a single ZIP file at http://hummod.org/baro-population.zip. Additionally, supplementary figures and instructions on how to run the experimental protocols can be found at: https://www.doi.org/10.6084/m9.figshare.16620073.v2.

In brief, organs and tissues that make up the model’s peripheral circulation include the kidneys, heart, skeletal muscle, gastrointestinal tract, liver, bone, brain, fat, skin, and lungs. Blood flow through these organ systems is determined by BP gradient and vascular resistance. These resistances can be modulated by the SNS, ANG II, and local tissue factors such as oxygen. Both kidneys in HumMod are separated into both vascular and tubular components. Renal hemodynamics and tubular functions are influenced by physical factors, SNS, hormones (e.g. ANG II, aldosterone, and ANP), and tubuloglomerular feedback (TGF). Physiological responses to ANP in the model were derived from studies conducted in both dogs and rats [26–33]. The physiological effects from RSNA are also derived from the animal literature [34]. Specific description of these relationships and equations can be found in the supplementary material and in previous work [11, 17]. Normal baseline values of these model variables are provided in Table 1.

**Table 1:** Cardiovascular, hormonal, and neural variables at normal model conditions and in the calibrated hypertensive virtual population (n = 60) before and after 1 month of baroreflex activation therapy.

Variable	Normal^a^	Baseline	BAT	%Change
MAP (mmHg)	92	115 ± 6	100 ± 5*	-14 ± 3
Heart Rate (bpm)	72	85 ± 19	75 ± 19*	-11 ± 3
Cardiac output (mL/min)	5.2	6147 ± 397	6087 ± 388*	-1 ± 2
Blood volume (mL)	5.7	5875 ± 315	6181 ± 237*	5 ± 3
ECF Volume (L)	15.4	16.4 ± 0.4	16.5 ± 0.4*	1 ± 1
TPR (mmhg/mL/min)	0.018	0.019 ± 0.001	0.016 ± 0.001*	-13 ± 4
Angiotensin II (pg/mL)	11	14 ± 4	10 ± 4*	-24 ± 16
Aldosterone (pMol/L)	272	272 ± 42	257 ± 47*	-6 ± 8
ANP (pMol/L)	25	24 ± 19	39 ± 23*	72 ± 44
Norepinephrine (pg/mL)	234	277 ± 32	211 ± 23*	-24 ± 2
Peripheral SNA (Hz)	1.5	1.8 ± 0.2	1.3 ± 0.2*	-26 ± 1
Cardiac SNA (Hz)	1.5	1.9 ± 0.3	1.3 ± 0.2*	-30 ± 3
Renal SNA (Hz)	1.5	2.4 ± 0.4	1.7 ± 0.3*	-29 ± 3
Fat mass (kg)	15	21.3 ± 0.4	21.6 ± 0.8	1 ± 1
Nephrons (million)	2.4	2.2 ± 0.2	2.2 ± 0.2	0 ± 0

BAT indicates baroreflex activation therapy; MAP, mean arterial pressure; TPR, total peripheral resistance; RAP, right atrial pressure; ANP, atrial natriuretic peptide; SNA, sympathetic nerve activity; GFR, glomerular filtration rate; and NaR, sodium reabsorption.

aDefault model values before constructing the hypertensive population.

*p < 0.05 vs. Baseline.

### Virtual population

First, models (uncalibrated) were created by randomly varying cardiovascular and renal model coefficients (n = 335) in a uniform way within 5% of their baseline default value, with the exception of coefficients with larger known physiological variability. These exceptions were salt intake (0.8x to 1.2x normal), baseline renal mass (0.8x – 1.2x normal), SNA to the heart (1x to 1.5x normal), SNA to the periphery (1x to 1.5x nor), SNA to the kidney (1x to 2x normal), aldosterone secretion (0.5x to 1.5x normal), renin secretion (1x to 2x normal), and baseline sinoatrial rhythm (0.75x to 1.5x normal), ranges similar to findings seen in obese humans with HTN [35–37]. Additional model changes to mimic human obesity included increases in fat mass 1.3 to 1.5x normal and increases in fractional proximal tubular sodium reabsorption (PTNaR) 1.1x to 1.4x normal [38]. After coefficient and distribution designation, an initial population was created by sampling these independent distributions 7000 times for 7,000 models. After running each uncalibrated model, 6,092 models completed the BAT protocol and were subjected to a nearest-neighbor calibration algorithm using Mathematica^TM^ 11. A new calibrated set of coefficients were created, and the same BAT protocol was simulated with the new calibrated models.

### Calibration algorithm

Based on MAP, GFR, and PRA before and after BAT as coordinates, we created a 6-dimensional space. A parallelepiped in the space with side lengths equal to one standard deviation was centered at each experimental data point. For each animal (n = 6), 10 virtual patients within 1 standard deviation in each of the variable dimensions (n = 6) were sampled from the uncalibrated population. These selected virtual patients (n = 60) were associated with unique coefficient sets. We centered a uniform kernel at each patient’s location in the coefficient space and combined these kernels into a mixture (additive) distribution. The new mixture distribution replaced the original uniform distribution for sampling a new virtual population. Re-varying the coefficient sets of these selected virtual patients (up to 2.5%) yielded a new population of models that we term a calibrated virtual population.

### Simulation protocol

Models were simulated with water ad libitum, constant food intake, and constant sodium intake throughout the simulation (139 ± 18 mmol/day). All models (both uncalibrated and calibrated) were run for a 4-week baseline to achieve steady-state values. After steady-state was achieved, BAT was simulated in the model by increasing carotid baroreceptor input into the central nervous system to 40% above normal. This level of afferent input was held constant for 4 weeks in all simulations.

In addition to BAT alone (BAT Control), calibrated models were used for additional simulations during BAT. These included: BAT with ANP clamped at baseline levels (ANP Clamp), BAT with the baroreflex input to the kidney clamped at baseline levels (RSNA Clamp), and the combination of both factors clamped at baseline (ANP+RSNA). Outputs of all simulations, unless stated otherwise, include baseline and 1 hour (immediate) and 4 weeks after BAT.

### Statistical analysis

Data were summarized using mean ± standard deviations. Data was analyzed with unpaired t-tests (two-sided) with Welch’s correction or paired t-tests as appropriate. When assumptions of normality were not justified, the data were also analyzed with the nonparametric Mann-Whitney test or Wilcoxon nonparametric signed rank test with paired data. For analyses of variables over time (Figs 3–6), two-way ANOVA repeated measures was used followed by Tukey’s post hoc test. The nonparametric Spearman test was used to detect significant correlations among baseline variables with the fall in BP. All statistical analyses were performed using GraphPad Prism 8 (La Jolla, CA). Probability was based on two-tailed tests of significance, and significance was considered p<0.05.

## Results

**Fig 1:**
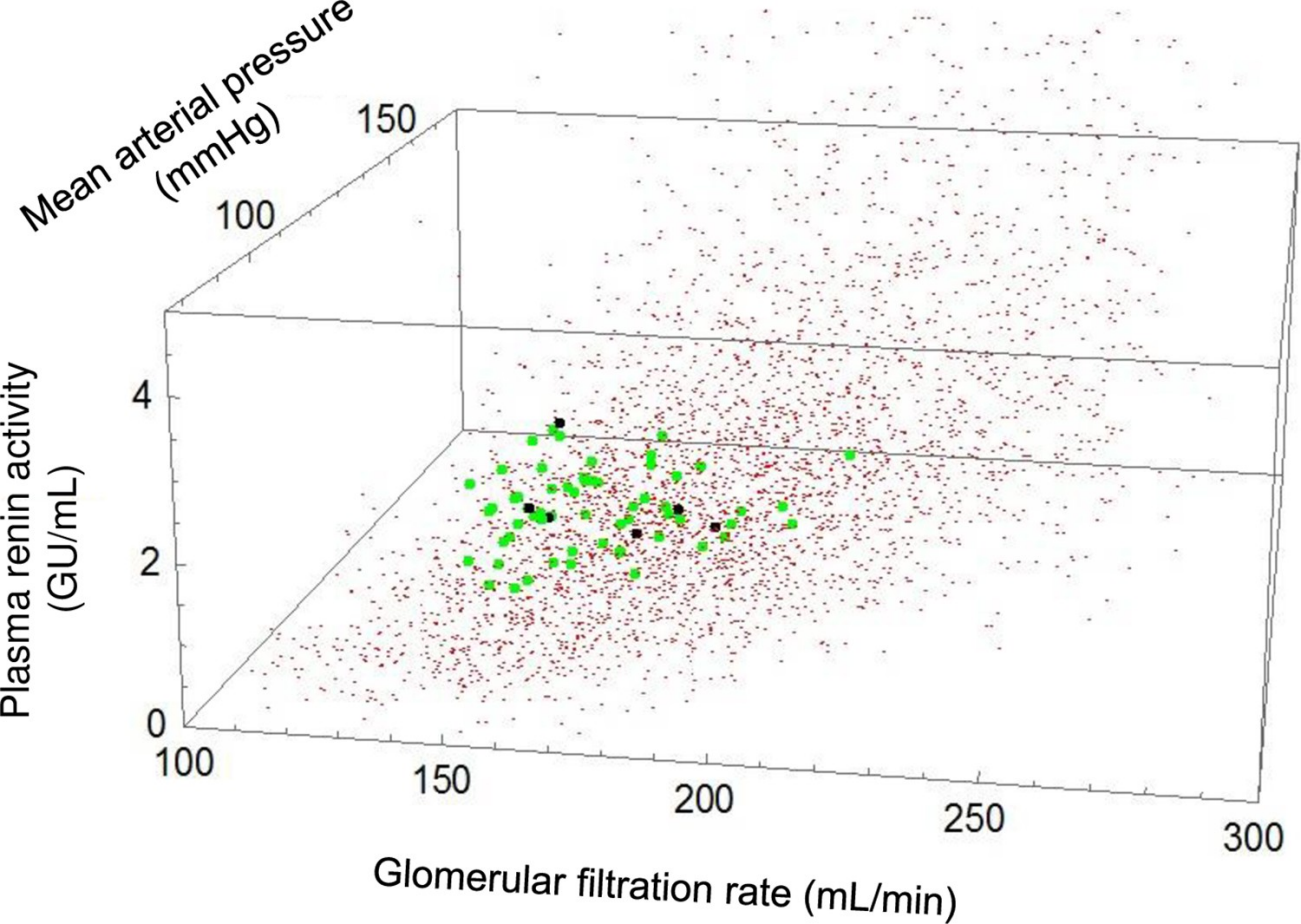
Baseline variables in the experimental (n = 6, shown in black from [22]) and virtual populations (both uncalibrated [n = 6,092 in red] and calibrated [n = 60, in green]) in three dimensions: Mean arterial pressure, plasma renin activity, and glomerular filtration rate.

**Fig 2:**
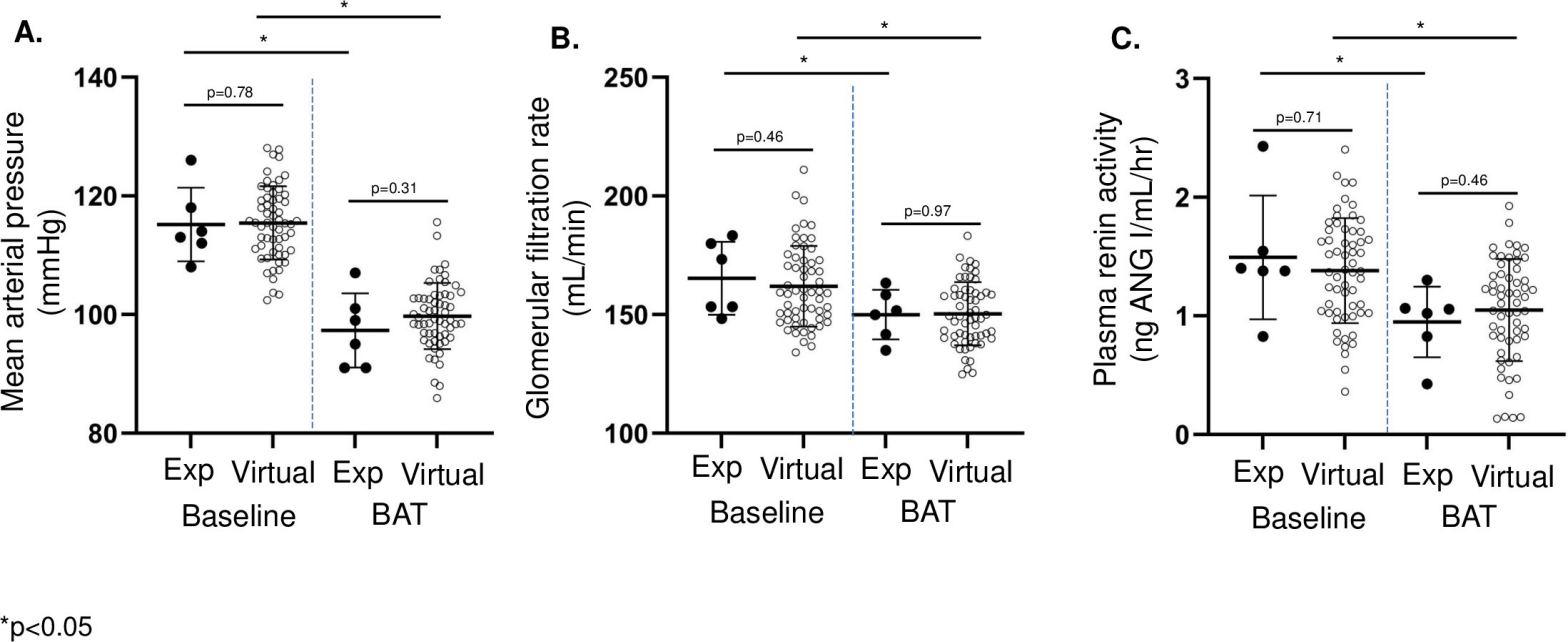
Responses to BAT in experimental [22] (Exp) and virtual populations. Mean arterial pressure, glomerular filtration rate, and plasma renin activity were not significantly different between the two groups at baseline or after BAT.

Random generation of models with 5% parameter variability resulted in 6,092 initial models (Fig 1 shown in red). Calibration resulted in a virtual population (n = 60, shown in green) with similar baseline MAP, GFR, and PRA as experimental data (n = 6, shown in black, Fig 1). Most significantly, there were comparable decreases in MAP, GFR, and PRA during BAT in the dogs and virtual populations (Fig 2).

Additional cardiovascular, neurohormonal, and renal responses to BAT in the virtual calibrated population are illustrated in Tables 1 and 2. At baseline, the virtual population presented with HTN, increased cardiac output, increased circulating levels of ANG II and aldosterone, glomerular hyperfiltration, increased renal sodium reabsorption, and greater peripheral and renal SNA relative to normal (Tables 1 and 2), similar to humans with obesity-mediated HTN [3, 24, 35, 36]. Baseline HTN in the virtual population was associated with higher-than-normal PTNaR (Table 2), primarily due to elevated RSNA and ANG II (Table 2). MAP, heart rate, and sympathetic outflow to the periphery, heart, and kidney were significantly reduced after four weeks of BAT (Table 1). Additionally, during BAT, there were significant increases in blood volume (6181 ± 237 vs. 5875 ± 315 mL, respectively), right atrial pressure (1.4 ± 0.6 vs. 1.0 ± 0.6 mL, mmHg), and circulating ANP (39 ± 23 vs. 24 ± 19 pMol/L, respectively) as compared to baseline. The effects of BAT on renin secretion and PTNaR were primarily through reductions in RSNA. More specifically, in response to BAT the β_1_- adrenergic effect on renin secretion decreased (1.0 ± 0.1 vs. 1.4 ± 0.3) and the α_1_- adrenergic effect on PTNaR decreased (1.3 ± 0.1 vs. 1.4 ± 0.1) significantly as compared to baseline (Table 2). The BAT-induced change in afferent arteriolar resistance was mainly through effects on tubuloglomerular (TGF) feedback (Table 2).

**Table 2:** Renal responses at normal model conditions and in the calibrated hypertensive virtual population before and after 1 month of baroreflex activation therapy.

Variable	Normal^a^	Baseline	BAT	%Change
GFR (mmHg)	123	162 ± 17	150 ± 13	-7 ± 4*
Renal blood flow (mL/min)	1025	1012 ± 272	1146 ± 221	3 ± 5*
Total NaR (mmol/min)	16.6	22 ± 3	21 ± 2	-7 ± 4*
Renin Secretion (mmol/min)	80	109 ± 35	84 ± 36	-25 ± 18*
Macula Densa Effect (xNormal)		1.09 ± 0.29	1.13 ± 0.36	2 ± 15*
β_1_-Adrenergic Effect (xNormal)		1.43 ± 0.25	1.02 ± 0.14	-28 ± 5*
PT NaR (mmol/min)	10	16 ± 2	15 ± 2	-8 ± 5*
ANG II Effect (xNormal)		1.14 ± 0.18	1.03 ± 0.17	-10 ± 6*
SNA Effect (xNormal)		1.44 ± 0.09	1.31 ± 0.08	-9 ± 4*
ANP Effect (xNormal)		1.01 ± 0.05	0.97 ± 0.09	-4 ± 2*
IFP Effect (xNormal)		0.79 ± 0.06	0.98 ± 0.07	23 ± 7*
AA resistance (mmhg/mL/min)	0.063	0.075 ± 0.01	0.055 ± 0.01	-26 ± 6*
TGF Effect (xNormal)		1.04 ± 0.16	0.83 ± 0.19	-24 ± 7*
Viscosity Effect (xNormal)		0.98 ± 0.04	0.93 ± 0.03	-6 ± 3*
ANP Effect (xNormal)		0.98 ± 0.04	0.97 ± 0.06	-1 ± 1*
SNA Effect (xNormal)		1.02 ± 0.12	1.00 ± 0.13	-2 ± 3*
Myogenic Effect (xNormal)		1.00 ± 0.002	1.00 ± 0.002	0 ± 0

Effects are multipliers on PT NaR and AA resistance. Normal model values for effect multipliers have a default value of 1. BAT indicates baroreflex activation therapy; GFR, glomerular filtration rate; NaR, sodium reabsorption; PT, proximal tubule; ANG II, angiotensin II; SNA, sympathetic nerve activity; ANP, atrial natriuretic peptide; IFP, interstitial fluid pressure; AA, afferent arteriole; TGF, tubuloglomerular feedback; and RSNA, renal sympathetic nerve activity.

*p < 0.05 Baseline vs. BAT.

^a^Default model values before constructing the hypertensive population.

Table 3 provides Spearman correlations between the BP change after BAT and several baseline variables describing SNA, renal function, hormonal control, systemic hemodynamics, and systemic volumes. These baseline variables describe a virtual patient that would most likely respond to BAT, which included models with high SNA, increased GFR and nephron mass, elevated tubular sodium reabsorption, low plasma ANP, increased sensitivities to ANP and ANG II, increased TPR and MAP, and lower systemic volumes (Table 3). Physiological relationships including baroreflex sensitivity, PTNaR, plasma ANG II and aldosterone, and salt intake were not significantly correlated with the BAT-induced BP fall (Table 3).

**Table 3:** Correlations among baseline variables and the change in mean blood pressure.

Baseline Variable	r	95% CI	p-value
**SNA**			
Cardiac SNA	-0.70	-0.82 to -0.54	<0.0001
Epinephrine	-0.63	-0.76 to -0.43	<0.0001
Peripheral SNA	-0.51	-0.68 to -0.28	<0.0001
β_1_-adrenergic effect (renin secretion)	-0.50	-0.67 to -0.27	<0.0001
SNA effect (PTNaR)	-0.49	-0.67 to -0.26	<0.0001
Norepinephrine	-0.48	-0.66 to -0.25	0.0001
Renal SNA	-0.45	-0.64 to -0.21	0.0003
Atrial receptor activity	0.46	0.22 to 0.64	0.0003
SNA effect (Aff art)	0.16	-0.11 to 0.40	0.2395
Baroreflex Sensitivity	-0.22	-0.45 to 0.04	0.09
**Renal**			
CDNaR (%)	-0.59	-0.74 to -0.38	<0.0001
Nephron count	-0.40	-0.60 to -0.15	0.0019
MD Na+ effect (renin secretion)	0.38	0.13 to 0.58	0.0033
CDNaR	-0.36	-0.57 to -0.11	0.0052
Loop NaR	-0.30	-0.52 to -0.04	0.0198
DTNaR	-0.28	-0.50 to -0.02	0.0328
GFR	-0.27	-0.49 to -0.002	0.0424
PTNaR	-0.15	-0.40 to 0.12	0.2535
**Hormonal**			
ANP effect (PTNaR)	-0.61	-0.75 to -0.41	<0.0001
Plasma ANP	0.68	0.50 to 0.80	<0.0001
ANP Effect (Aff art Conductance)	0.40	0.16 to 0.60	0.0015
ANG II Effect (PTNaR)	0.32	0.06 to 0.54	0.0137
Renin secretion rate	0.28	0.01 to 0.50	0.0349
ANG II	0.22	-0.05 to 0.46	0.0975
Aldosterone	0.10	-0.17 to 0.35	0.4516
**Systemic hemodynamics and volumes**			
TPR	-0.58	-0.73 to -0.37	<0.0001
Right atrial pressure	0.52	0.30 to 0.69	<0.0001
Plasma volume	0.55	0.33 to 0.71	<0.0001
Blood volume	0.55	0.34 to 0.71	<0.0001
Systemic vein volume	0.55	0.34 to 0.71	<0.0001
HR	-0.48	-0.66 to -0.24	0.0001
MAP	-0.47	-0.65 to -0.24	0.0002
Left atrial pressure	0.44	0.20 to 0.63	0.0006
Mean arterial pressure	-0.41	-0.60 to -0.16	0.0014
ECFV	0.33	0.07 to 0.54	0.0113
ECF Na+	0.26	0.0004 to 0.49	0.0436
Sodium Intake	-0.08	-0.33 to 0.18	0.5588

Nonparametric Spearman correlation coeefficients, 95% CI, and cooresponding p-values are given. Sodium reabsorption variables indicate absolute reabsorption unless otherwise noted. SNA indicates sympathetic nerve activity; PTNaR, proximal tubular sodium reabsorption; Aff art, afferent artery; CDNaR, collecting duct sodium reabsorption; MD, macula densa; DTNaR, distal tubular sodium reabsorption; GFR, glomerular filtration rate; ANP, atrial natriuretic peptide; ANG II, angiotensin II; TPR, total peripheral resistance; MAP, mean arterial pressure; HR, heart rate; ECFV, extracellular fluid volume; and ECF, extracellular fluid.

**Fig 3:**
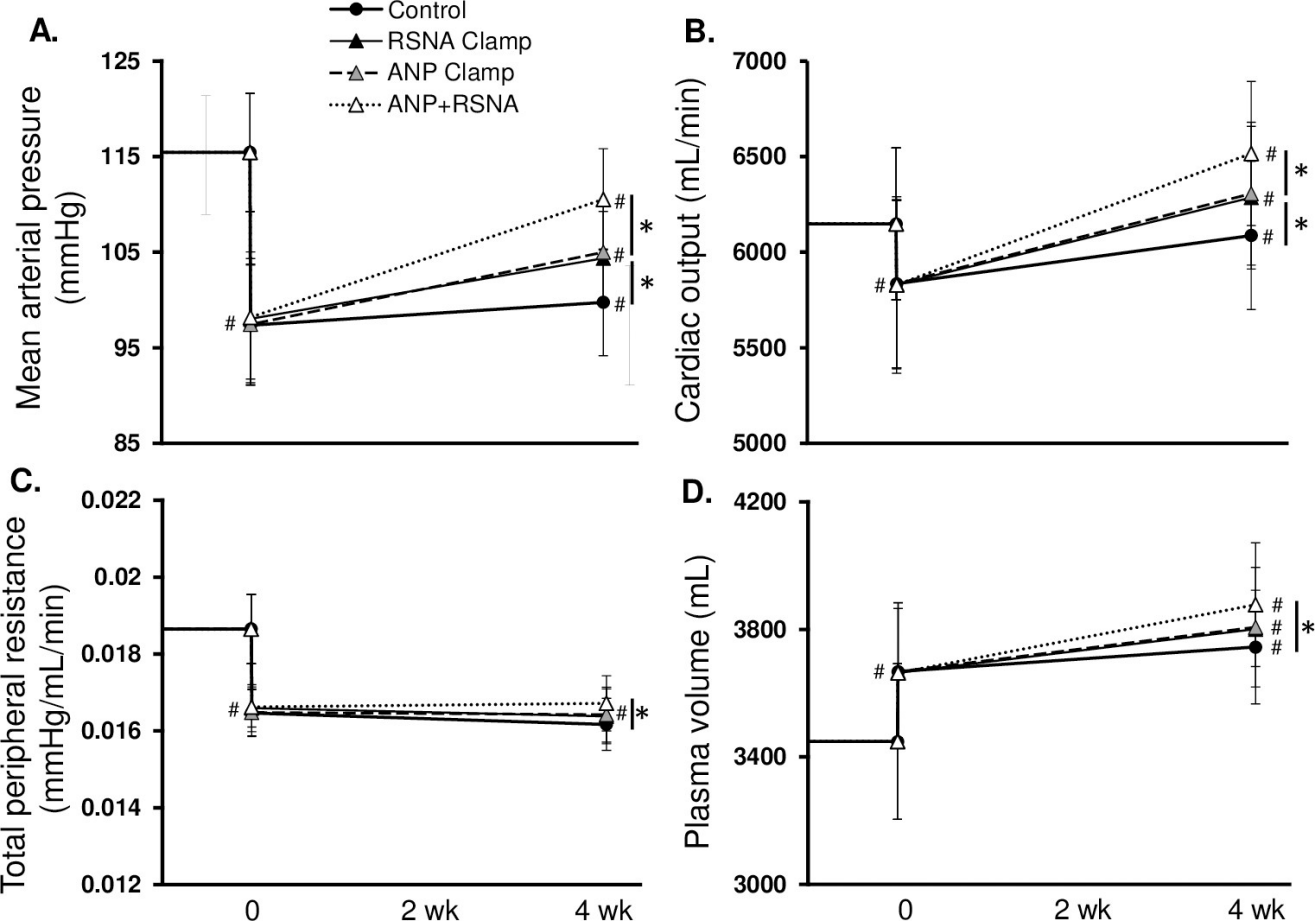
Cardiovascular responses to BAT among control (BAT Control), RSNA Clamp, ANP Clamp, and ANP+RSNA Clamp simulations. *p<0.05; #p<0.05 vs. baseline.

**Fig 4:**
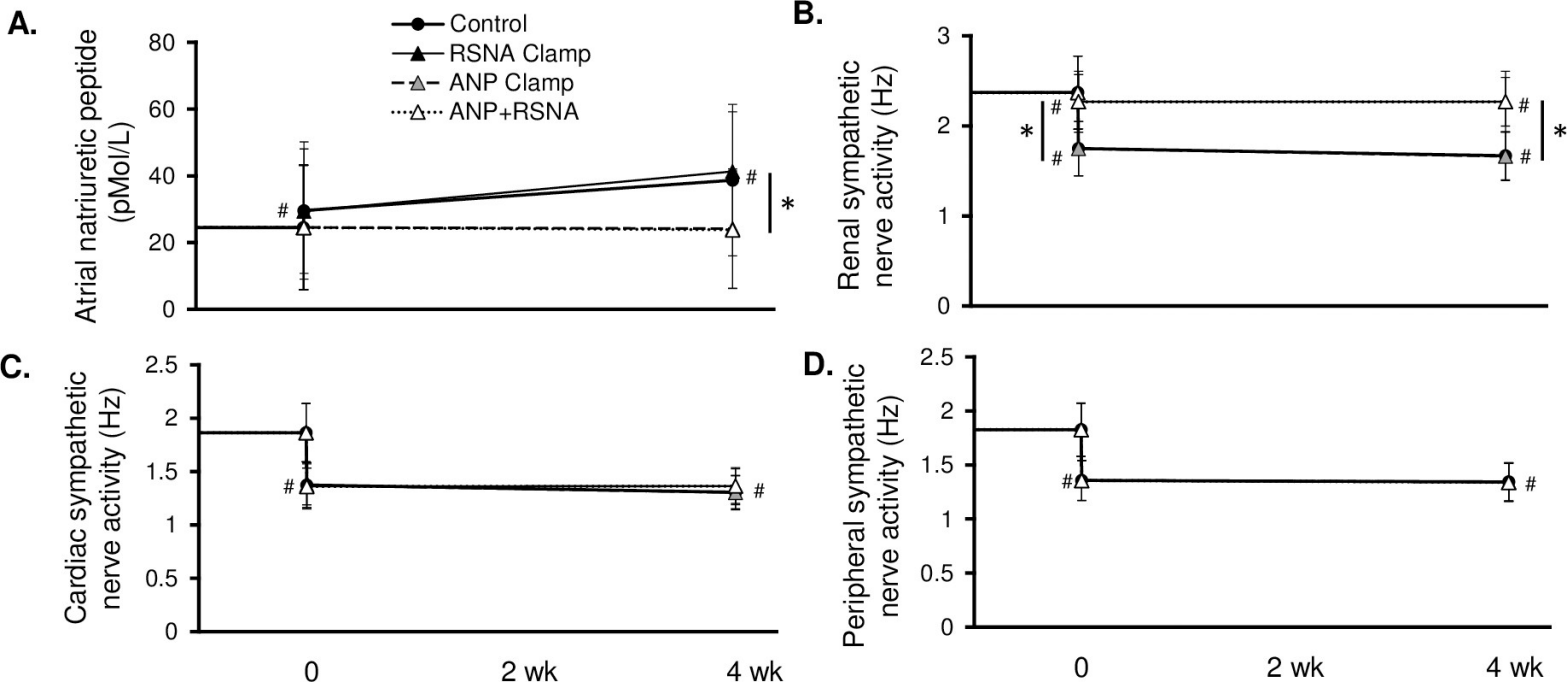
Atrial natriuretic peptide (ANP), renal sympathetic nerve activity (RSNA), cardiac sympathetic nerve activity, and peripheral sympathetic nerve activity during 1 month of BAT in the control, RSNA Clamp, ANP Clamp, and ANP+RSNA Clamp simulations. *p<0.05; #p<0.05 vs. baseline.

**Fig 5:**
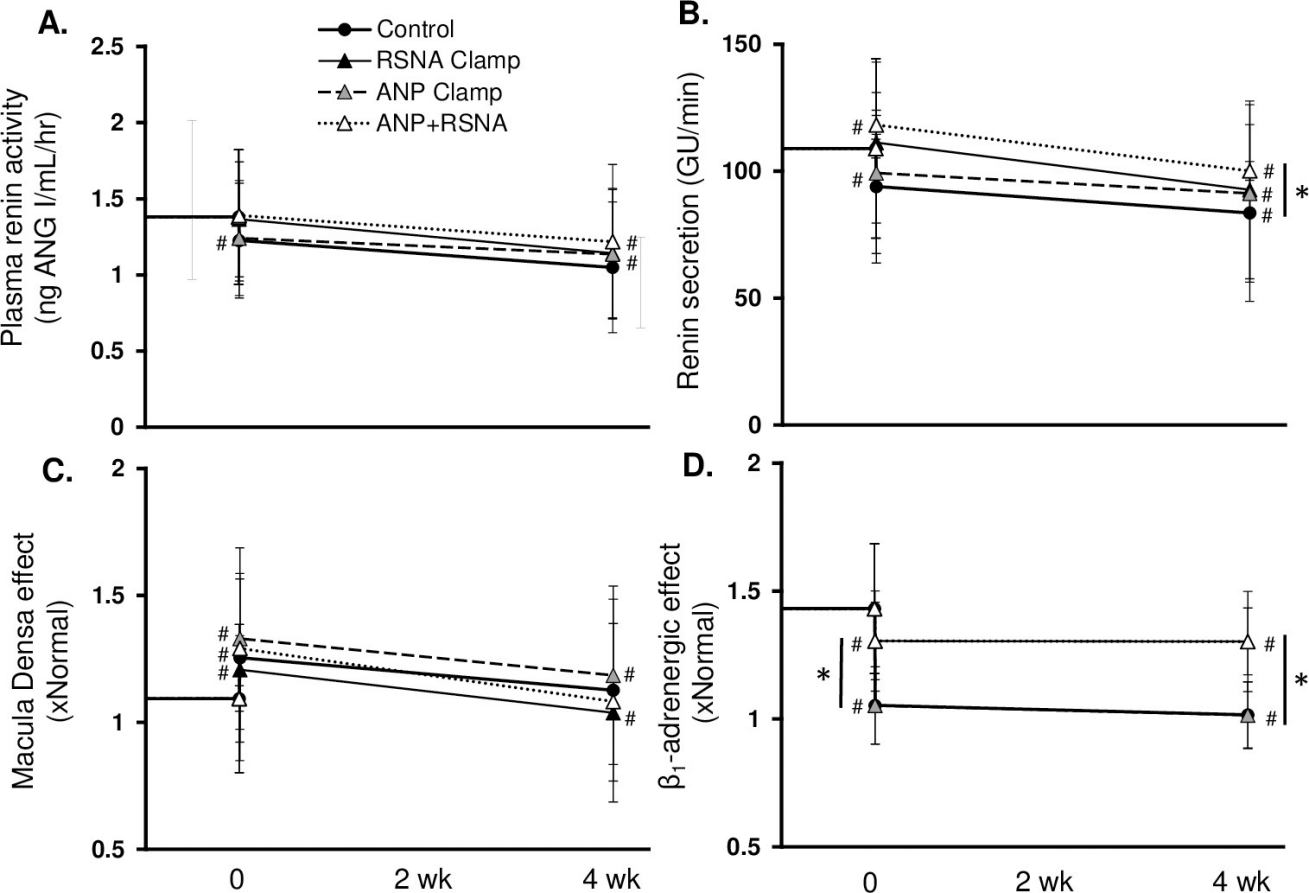
Renin secretion and its determinants before and during BAT in the control, RSNA Clamp, ANP Clamp, and ANP+RSNA Clamp simulations. *p<0.05; #p<0.05 vs. baseline.

**Fig 6:**
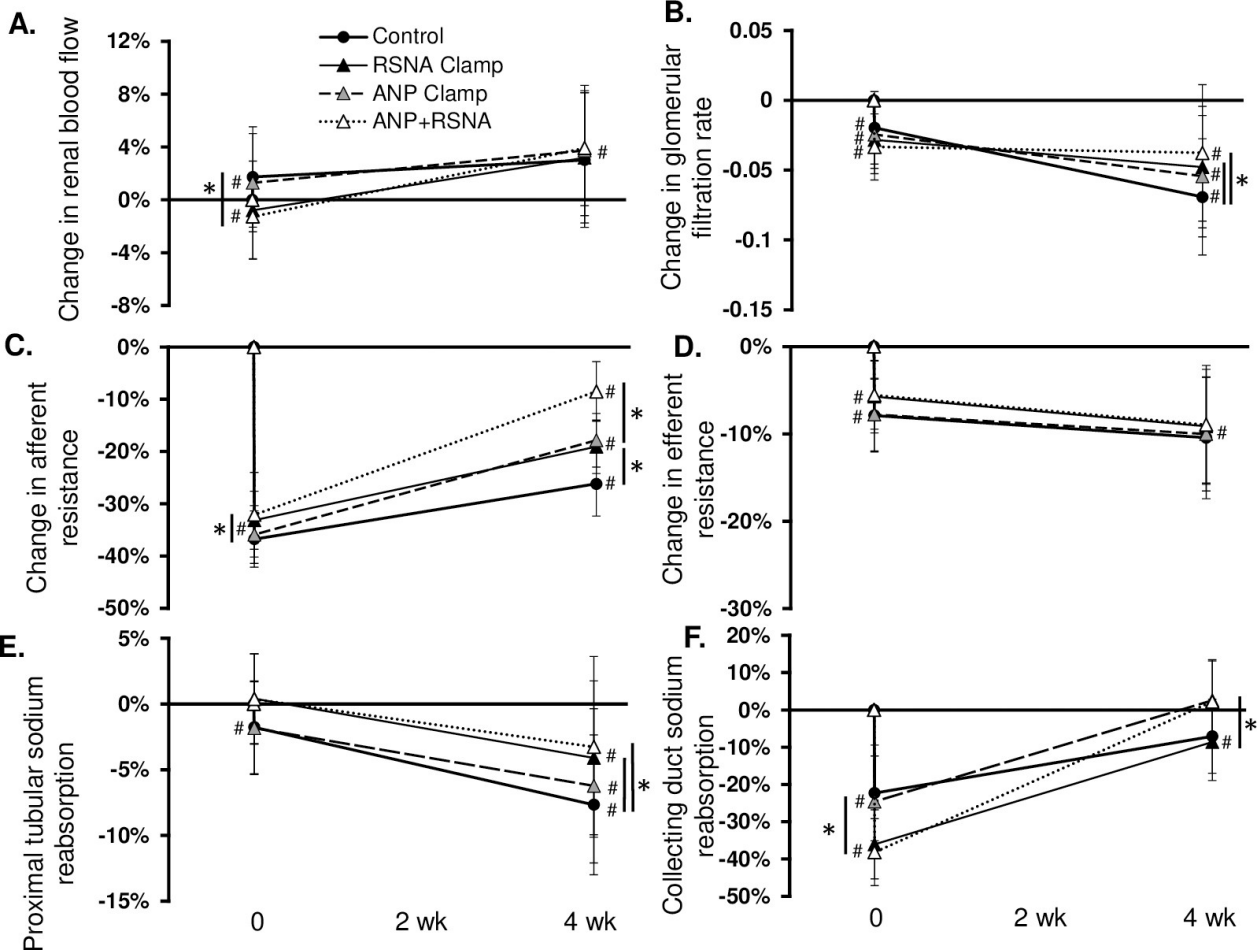
Changes in renal function during BAT in the control, RSNA Clamp, ANP Clamp, and ANP+RSNA Clamp simulations. *p<0.05; #p<0.05 vs. baseline.

The relative roles of ANP and RSNA on the chronic physiological responses to BAT are shown in Figs 3–6. Most significantly, as compared to the 16 mmHg reduction in MAP after BAT in the control simulation, the fall in BP was significantly less with the ANP (10.4 ± 4 mmHg), the RSNA (11.1 ± 5 mmHg), and the combination clamps (4.9 ± 4 mmHg) (Fig 3). When ANP, RSNA, plasma ANG II and catecholamines were clamped at baseline, the BAT-induced reduction in MAP was completely abolished (S1 Fig). Immediately after BAT in all simulations TPR and CO decreased while plasma volume increased. Under chronic conditions, TPR remained depressed while CO increased to control levels along with further expansion of PV. Fig 4 illustrates changes in ANP, cardiac SNA, RSNA, and peripheral SNA in the 4 simulations. Of greatest relevance, plasma ANP and RSNA remained at similar levels as baseline during their respective clamps (Fig 4).

Plasma renin activity and renin secretion decreased significantly during chronic BAT in all simulations (Fig 5). Chronic suppression of renin secretion occurred even in the RSNA clamped simulation due to a fall in stimulation of renal β_1_-adrenergic receptors associated with a significant fall in circulating catecholamines, which occurred in all simulations.

Finally, 1 month of BAT increased renal blood flow (RBF) and decreased GFR in all simulations (Fig 6). Although BAT immediately increased RBF in the BAT Control and ANP Clamp simulations, RBF significantly decreased in the RSNA Clamp simulations (Fig 6A). This was largely due to the absence of the immediate fall in ANG II; however, over the next month of BAT, RBF increased to 3–4% above baseline, regardless of a properly functioning ANP secretion or RSNA. The decrease in GFR and afferent arteriolar resistance was directly related to the fall in renal perfusion pressure as well as the extent of efferent arteriolar vasodilation (Fig 6). For example, in the ANP+RSNA Clamp group, efferent arteriolar resistance and MAP did not fall as much which resulted in significantly higher GFR (155 ± 13 mL/min) as compared to the BAT Control simulation (150 ± 13 mL/min). Further, the change in afferent arteriolar resistance reflected changes in TGF, which was primarily dependent upon macula densa sodium delivery and renal perfusion pressure (i.e. MAP). Clamping RSNA and/or ANP resulted in a significant increase in afferent resistance after 1 month of BAT relative to the control simulation (Fig 6C). Finally, PTNaR and collecting duct sodium reabsorption significantly decreased upon initiation of BAT and remained below baseline over the next month in the BAT control simulation (Fig 6E and 6F). As compared to control, this chronic decrease in sodium reabsorption was significantly blunted in the proximal tubule with RSNA clamping (-8 ± 5% vs. -4 ± 6%, respectively) and in the collecting duct by clamping ANP (-7 ± 10% vs. 3 ± 11%, respectively) (Fig 6).

## Discussion

BAT has been shown to significantly lower BP in many patients with uncontrolled hypertension. Unfortunately, the mechanisms that contribute to BP lowering are not well understood and, consequently, the patient characteristics that predict or correlate with favorable responses to this therapy are unclear. In the present study, we used an integrative mathematical model of human physiology, HumMod, to create virtual patient populations that included cardiovascular, renal, neurohormonal, and metabolic values similar to patients with obesity-related HTN. Then, we simulated a clinical trial with BAT. The results of this study: 1) provide further validation that HumMod accurately reproduces responses to physiological interventions and support the utility of using the virtual population approach to elucidate mechanistic insight into HTN therapy, 2) suggest several baseline physiological factors of importance that predict the antihypertensive response to BAT, 3) suggest that suppression of RSNA and increases in ANP secretion contribute but, independently, are not obligatory factors for BP lowering with BAT, unless both are blocked simultaneously.

There has been substantial experimental and clinical evidence for the past 20 years demonstrating the importance of the SNS in obesity-associated HTN [24, 39]. Additionally, essential HTN, sleep apnea, or chronic kidney disease also increases long-term BP in humans, at least in part, through increases in RSNA [40, 41]. Even in obese *normotensive* patients, peripheral SNA and RSNA are increased [35, 36] suggesting that other systems may be important in the protection against sympathetic-mediated HTN. The virtual population in the current study was associated with HTN, increased circulating levels of ANG II and aldosterone, increased GFR, and increased global and renal SNA, which is similar to HTN in obese humans before major losses in renal function [24, 35]. After BAT, obese patients have significant falls in BP, to the same extent as lean counterparts [42]. Obesity-meditated HTN in dogs is associated with elevated renin that is reduced to normal with BAT or the removal of renal nerves [22]. Further, chronic antagonism of the renin-angiotensin system effectively reduces BP in obese animals [43] and humans [44]. Although reductions in renin are not obligatory for the reduction in BP during BAT [45], our simulations suggest that the renin-angiotensin system may still play some role in the efficacy of BAT in obesity. In fact, the remaining -5 mmHg change in MAP in the ANP+RSNA Clamp simulation was largely due to the small but significant falls in plasma renin and circulating catecholamines. When these systems were clamped at baseline in addition to ANP and RSNA, MAP did not significantly change 1 month after BAT (S1 Fig).

When the BAT-induced suppression of RSNA was blocked, BP lowering was significantly blunted (by ~30%) (Fig 3A). In the BAT Control group, the suppression in RSNA during BAT played a significant role in the decrease in PTNaR that occurred (Table 2). This was through RSNA’s direct effects on PTNaR and indirectly through influences on ANG II. Interestingly, in hypertensive patients with heart failure with preserved ejection fraction (or HFpEF, a population known to have exaggerated increases in cardiac SNA and RSNA [46, 47]), BAT reduces BP more significantly than in hypertensive patients without HFpEF [48]. However, in pathological conditions where RSNA cannot be suppressed or if RSNA is not elevated at baseline (as what occurs in patients with previous renal denervation or in the elderly [49]), BAT may still be efficacious due to compensating effects from ANP. Indeed, BAT significantly reduces BP in patients with prior renal denervation [50, 51] and in patients >60 years old [48]. Interestingly, experimental results in renal denervated normotensive dogs demonstrated a 14% fall in mean BP during BAT [10]. Similarly, clinical results demonstrate a 8–15% fall in mean BP in hypertensive patients who were previously renal denervated and then subjected to 6–12 months of BAT [50, 51]. These data are comparable to our RSNA clamp simulation that was associated with a 10% fall in mean BP during BAT.

While ANP’s role during BAT was significant in our normotensive mathematical model [11], its role in the treatment of HTN with this device has never been formally investigated. Lohmeier et al. originally demonstrated ANP increases with this device and may serve as an antihypertensive mechanism when the renal nerves are not intact [10]. Our results indicate that without functional ANP, there’s a significant blunting of the chronic BP lowering of BAT as compared to control (-10 vs. -16 mmHg, respectively; Fig 3). This was through multiple mechanisms. During BAT in the control simulation, the 72 ± 4% increase in circulating ANP was due to the increases in atrial pressure associated with the slowing of HR, similar to the physiological effects from beta blockade [52]. This increase in ANP was associated with reduced PTNaR (-8 ± 5%), afferent arteriolar resistance (-26 ± 6%), TGF (-24 ± 7%), and renin secretion (-25 ± 18) (Table 2). Further, if ANP was not allowed to increase during BAT, these physiological changes were significantly blunted: PTNaR (-6 ± 8%), afferent arteriolar resistance (-18 ± 5%), TGF (-13 ± 6%), and renin secretion (-17 ± 17). Pharmacological therapies that block the degradation of ANP significantly increase circulating ANP in normal and hypertensive patients and significantly reduce BP in hypertensives [53, 54]. The importance of future clinical studies needed to confirm ANP’s role during BAT can not be overstated.

There was careful parameterization of this population model for the magnitude and range of SNA (cardiac, renal, and peripheral) that is seen in hypertensive obese humans [35–37] in order to increase the confidence in the predictions of BAT responses. However, there are limitations that need to be addressed. Based on the experimental data, all animals had a significant response during BAT (the change in MAP ranged from -11 to -27 mmHg). This high response rate could be particular to the specified population: young, obese, hypertensive dogs. Additionally, unlike clinical scenarios, experimental BAT studies usually have animals that are confined, environmental variables that are well controlled for, and continuously monitored BP [10, 22]. Clinical trials investigating BAT have previously targeted obese populations with resistant HTN and on multiple antihypertensive drugs with prevalences of comorbidities such as diabetes, heart failure, and CKD [42, 48]. Response rates to BAT in these populations has been shown to be as low as 55% [42]. These participants can vary widely in differing age, sex, and race that can affect underlying physiology and should be taken into consideration. Future simulations will focus on increasing the pathophysiology of the models, most likely resulting in increasing variability and increased resistance to this device. Although baroreceptor input to the kidney was fixed at baseline levels, slight influences from chemoreceptors and ANG II caused a small but significant fall in RSNA in the RSNA clamped simulations. Chemoreceptor activation, which occurs in obesity at baseline, may play a small role in BAT’s efficacy as shown by the decreased respiratory drive that occurs during BAT in obese dogs [55]. However, in our model, chemoreceptor input, along with ANG II and circulating catecholamines altogether accounted for minor changes (<5 mmHg) in the BP change during BAT. Whether or not this mechanism plays an important role in humans is unknown. Finally, physiological modeling should not replace experimental studies, but rather, serve as a tool to generate consistent explanations of mechanisms and test hypotheses that can improve future experimental design.

## Supporting information

S1 File (DOCX)

S1 Fig Cardiovascular and renal responses to BAT in a control simulation (BAT Control) and with several factors clamped at baseline. These factors included atrial natriuretic peptide (ANP), renal sympathetic nerve activity (RSNA), angiotensin II (ANG II), norepinephrine (NE), and epinephrine (Epi). *p<0.05; #p<0.05 vs. baseline.
(PDF)

S2 Fig Equations and model parameters for calculating glomerular filtration rate. FF indicates filtration fraction; SNGFR, single nephron glomerular filtration rate; PT_conductance_, conductance of the proximal tubule; RBF, renal blood flow; Kf, filtration coefficient; P_C_, capillary hydrostatic pressure; P_BC_, Bowman’s Capsule hydrostatic pressure; P_osm_, capillary colloid osmotic pressure; and RPF, renal plasma flow. *Indicates implicit equation.
(PDF)

S3 Fig Peripheral blood flow and determinants of organ conductances in the model. Ang II indicates angiotensin II; SM, skeletal muscle; PO_2_, partial pressure of oxygen; ADH, antidiuretic hormone; GI, gastrointestinal; PCO_2_, partial pressure of carbon dioxide; and temp, temperature. *Indicates a negative relationship.
(PDF)

S4 Fig Determinants of afferent and efferent arteriolar conductance in the model. Symp indicates sympathetic; ANP, atrial natriuretic peptide; myo, myogenic; TGF, tubuloglomerular feedback; CCB, calcium channel blocker; and Ang II, angiotensin II. Data point on each relationship indicates normal model values. *Myogenic effect resets within 12 hours.
(PDF)

S5 Fig Determinants of tubuloglomerular feedback. Ang II indicates angiotensin II; and ANP, atrial natriuretic peptide. (PDF)

S6 Fig Determinants of renin secretion in the model. MD indicates macula densa; symp, sympathetic; and ANP, atrial natriuretic peptide.
(PDF)

S7 Fig Body compartment volumes. (PDF)

S8 Fig Determinants of proximal tubular sodium reabsorption. PTNa indicates proximal tubular sodium; angiotensin II, Ang II; sympathetic, symp; atrial natriuretic peptide, ANP; and renal interstitial fluid pressure, RIFP.
(PDF)

S9 Fig Determinants of loop of henle sodium reabsorption. Single nephron glomerular filtration rate, SNGFR; aldosterone, Aldo.
(PDF)

S10 Fig Determinants of distal tubular sodium reabsorption. Single nephron, SN; aldosterone, Aldo.
(PDF)

S11 Fig Determinants of collecting duct sodium reabsorption. Single nephron, SN; aldosterone, Aldo.
(PDF)
