# Supplementary material for: In silico trial of baroreflex activation therapy for the treatment of obesity-induced hypertension
Source: PLoS One. 2021 Nov 18;16(11):e0259917. doi: 10.1371/journal.pone.0259917 (PMC8601446; doi:10.1371/journal.pone.0259917)
Supplement: S1 File — (DOCX) [file pone.0259917.s001.docx]

**BibTeX References**

@article{RN9,

author = {Bisognano, J. D. and Bakris, G. and Nadim, M. K. and Sanchez, L. and Kroon, A. A. and Schafer, J. and de Leeuw, P. W. and Sica, D. A.},

title = {Baroreflex activation therapy lowers blood pressure in patients with resistant hypertension: results from the double-blind, randomized, placebo-controlled rheos pivotal trial},

journal = {J Am Coll Cardiol},

volume = {58},

number = {7},

pages = {765-73},

ISSN = {1558-3597 (Electronic)

0735-1097 (Linking)},

DOI = {10.1016/j.jacc.2011.06.008},

url = {<http://www.ncbi.nlm.nih.gov/pubmed/21816315>},

year = {2011},

type = {Journal Article}

}

@article{RN43,

author = {Boustany, C. M. and Brown, D. R. and Randall, D. C. and Cassis, L. A.},

title = {AT1-receptor antagonism reverses the blood pressure elevation associated with diet-induced obesity},

journal = {Am J Physiol Regul Integr Comp Physiol},

volume = {289},

number = {1},

pages = {R181-6},

ISSN = {0363-6119 (Print)

0363-6119 (Linking)},

DOI = {10.1152/ajpregu.00507.2004},

url = {<http://www.ncbi.nlm.nih.gov/pubmed/15774768>},

year = {2005},

type = {Journal Article}

}

@article{RN33,

author = {Brenner, B. M. and Ballermann, B. J. and Gunning, M. E. and Zeidel, M. L.},

title = {Diverse biological actions of atrial natriuretic peptide},

journal = {Physiol Rev},

volume = {70},

number = {3},

pages = {665-99},

ISSN = {0031-9333 (Print)

0031-9333 (Linking)},

DOI = {10.1152/physrev.1990.70.3.665},

url = {<http://www.ncbi.nlm.nih.gov/pubmed/2141944>},

year = {1990},

type = {Journal Article}

}

@article{RN2,

author = {Carey, R. M. and Calhoun, D. A. and Bakris, G. L. and Brook, R. D. and Daugherty, S. L. and Dennison-Himmelfarb, C. R. and Egan, B. M. and Flack, J. M. and Gidding, S. S. and Judd, E. and Lackland, D. T. and Laffer, C. L. and Newton-Cheh, C. and Smith, S. M. and Taler, S. J. and Textor, S. C. and Turan, T. N. and White, W. B. and American Heart Association Professional/Public, Education and Publications Committee of the Council on, Hypertension and Council on, Cardiovascular and Stroke, Nursing and Council on Clinical, Cardiology and Council on, Genomic and Precision, Medicine and Council on Peripheral Vascular, Disease and Council on Quality of, Care and Outcomes, Research and Stroke, Council},

title = {Resistant Hypertension: Detection, Evaluation, and Management: A Scientific Statement From the American Heart Association},

journal = {Hypertension},

volume = {72},

number = {5},

pages = {e53-e90},

ISSN = {1524-4563 (Electronic)

0194-911X (Linking)},

DOI = {10.1161/HYP.0000000000000084},

url = {<http://www.ncbi.nlm.nih.gov/pubmed/30354828>},

year = {2018},

type = {Journal Article}

}

@article{RN38,

author = {Chagnac, A. and Herman, M. and Zingerman, B. and Erman, A. and Rozen-Zvi, B. and Hirsh, J. and Gafter, U.},

title = {Obesity-induced glomerular hyperfiltration: its involvement in the pathogenesis of tubular sodium reabsorption},

journal = {Nephrol Dial Transplant},

volume = {23},

number = {12},

pages = {3946-52},

ISSN = {1460-2385 (Electronic)

0931-0509 (Linking)},

DOI = {10.1093/ndt/gfn379},

url = {<http://www.ncbi.nlm.nih.gov/pubmed/18622024>},

year = {2008},

type = {Journal Article}

}

@article{RN21,

author = {Clemmer, J. S. and Hester, R. L. and Pruett, W. A.},

title = {Simulating a virtual population's sensitivity to salt and uninephrectomy},

journal = {Interface Focus},

volume = {8},

number = {1},

pages = {20160134},

ISSN = {2042-8898 (Print)

2042-8898 (Linking)},

DOI = {10.1098/rsfs.2016.0134},

url = {<http://www.ncbi.nlm.nih.gov/pubmed/29285341>},

year = {2018},

type = {Journal Article}

}

@article{RN17,

author = {Clemmer, J. S. and Pruett, W. A. and Coleman, T. G. and Hall, J. E. and Hester, R. L.},

title = {Mechanisms of blood pressure salt sensitivity: new insights from mathematical modeling},

journal = {Am J Physiol Regul Integr Comp Physiol},

volume = {312},

number = {4},

pages = {R451-R466},

ISSN = {1522-1490 (Electronic)

0363-6119 (Linking)},

DOI = {10.1152/ajpregu.00353.2016},

url = {<http://www.ncbi.nlm.nih.gov/pubmed/27974315>},

year = {2017},

type = {Journal Article}

}

@article{RN11,

author = {Clemmer, J. S. and Pruett, W. A. and Hester, R. L. and Iliescu, R. and Lohmeier, T. E.},

title = {Role of the Heart in Blood Pressure Lowering During Chronic Baroreflex Activation: Insight from an in Silico Analysis},

journal = {Am J Physiol Heart Circ Physiol},

ISSN = {1522-1539 (Electronic)

0363-6135 (Linking)},

DOI = {10.1152/ajpheart.00302.2018},

url = {<http://www.ncbi.nlm.nih.gov/pubmed/30004810>},

year = {2018},

type = {Journal Article}

}

@article{RN1,

author = {Collaborators, G. B. D. Risk Factor},

title = {Global, regional, and national comparative risk assessment of 84 behavioural, environmental and occupational, and metabolic risks or clusters of risks for 195 countries and territories, 1990-2017: a systematic analysis for the Global Burden of Disease Study 2017},

journal = {Lancet},

volume = {392},

number = {10159},

pages = {1923-1994},

ISSN = {1474-547X (Electronic)

0140-6736 (Linking)},

DOI = {10.1016/S0140-6736(18)32225-6},

url = {<http://www.ncbi.nlm.nih.gov/pubmed/30496105>},

year = {2018},

type = {Journal Article}

}

@article{RN52,

author = {Davis, M. E. and Richards, A. M. and Nicholls, M. G. and Yandle, T. G. and Frampton, C. M. and Troughton, R. W.},

title = {Introduction of metoprolol increases plasma B-type cardiac natriuretic peptides in mild, stable heart failure},

journal = {Circulation},

volume = {113},

number = {7},

pages = {977-85},

ISSN = {1524-4539 (Electronic)

0009-7322 (Linking)},

DOI = {10.1161/CIRCULATIONAHA.105.567727},

url = {<http://www.ncbi.nlm.nih.gov/pubmed/16476851>},

year = {2006},

type = {Journal Article}

}

@article{RN48,

author = {de Leeuw, P. W. and Bisognano, J. D. and Bakris, G. L. and Nadim, M. K. and Haller, H. and Kroon, A. A. and HT, D. EBuT and Rheos Trial, Investigators},

title = {Sustained Reduction of Blood Pressure With Baroreceptor Activation Therapy: Results of the 6-Year Open Follow-Up},

journal = {Hypertension},

volume = {69},

number = {5},

pages = {836-843},

ISSN = {1524-4563 (Electronic)

0194-911X (Linking)},

DOI = {10.1161/HYPERTENSIONAHA.117.09086},

url = {<http://www.ncbi.nlm.nih.gov/pubmed/28320856>},

year = {2017},

type = {Journal Article}

}

@article{RN34,

author = {DiBona, G. F. and Kopp, U. C.},

title = {Neural control of renal function},

journal = {Physiol Rev},

volume = {77},

number = {1},

pages = {75-197},

ISSN = {0031-9333 (Print)

0031-9333 (Linking)},

DOI = {10.1152/physrev.1997.77.1.75},

url = {<http://www.ncbi.nlm.nih.gov/pubmed/9016301>},

year = {1997},

type = {Journal Article}

}

@article{RN49,

author = {Esler, M. and Jennings, G. and Biviano, B. and Lambert, G. and Hasking, G.},

title = {Mechanism of elevated plasma noradrenaline in the course of essential hypertension},

journal = {J Cardiovasc Pharmacol},

volume = {8 Suppl 5},

pages = {S39-43},

ISSN = {0160-2446 (Print)

0160-2446 (Linking)},

url = {<http://www.ncbi.nlm.nih.gov/pubmed/2427882>},

year = {1986},

type = {Journal Article}

}

@article{RN4,

author = {Esler, M. and Jennings, G. and Lambert, G. and Meredith, I. and Horne, M. and Eisenhofer, G.},

title = {Overflow of catecholamine neurotransmitters to the circulation: source, fate, and functions},

journal = {Physiol Rev},

volume = {70},

number = {4},

pages = {963-85},

ISSN = {0031-9333 (Print)

0031-9333 (Linking)},

DOI = {10.1152/physrev.1990.70.4.963},

url = {<http://www.ncbi.nlm.nih.gov/pubmed/1977182>},

year = {1990},

type = {Journal Article}

}

@article{RN39,

author = {Esler, M. and Rumantir, M. and Kaye, D. and Lambert, G.},

title = {The sympathetic neurobiology of essential hypertension: disparate influences of obesity, stress, and noradrenaline transporter dysfunction?},

journal = {Am J Hypertens},

volume = {14},

number = {6 Pt 2},

pages = {139S-146S},

ISSN = {0895-7061 (Print)

0895-7061 (Linking)},

DOI = {10.1016/s0895-7061(01)02081-7},

url = {<http://www.ncbi.nlm.nih.gov/pubmed/11411749>},

year = {2001},

type = {Journal Article}

}

@article{RN3,

author = {Grassi, G. and Mark, A. and Esler, M.},

title = {The sympathetic nervous system alterations in human hypertension},

journal = {Circ Res},

volume = {116},

number = {6},

pages = {976-90},

ISSN = {1524-4571 (Electronic)

0009-7330 (Linking)},

DOI = {10.1161/CIRCRESAHA.116.303604},

url = {<http://www.ncbi.nlm.nih.gov/pubmed/25767284>},

year = {2015},

type = {Journal Article}

}

@article{RN47,

author = {Grassi, G. and Quarti-Trevano, F. and Esler, M. D.},

title = {Sympathetic activation in congestive heart failure: an updated overview},

journal = {Heart Fail Rev},

volume = {26},

number = {1},

pages = {173-182},

ISSN = {1573-7322 (Electronic)

1382-4147 (Linking)},

DOI = {10.1007/s10741-019-09901-2},

url = {<http://www.ncbi.nlm.nih.gov/pubmed/31832833>},

year = {2021},

type = {Journal Article}

}

@article{RN37,

author = {Grassi, G. and Seravalle, G. and Colombo, M. and Bolla, G. and Cattaneo, B. M. and Cavagnini, F. and Mancia, G.},

title = {Body weight reduction, sympathetic nerve traffic, and arterial baroreflex in obese normotensive humans},

journal = {Circulation},

volume = {97},

number = {20},

pages = {2037-42},

ISSN = {0009-7322 (Print)

0009-7322 (Linking)},

DOI = {10.1161/01.cir.97.20.2037},

url = {<http://www.ncbi.nlm.nih.gov/pubmed/9610534>},

year = {1998},

type = {Journal Article}

}

@article{RN46,

author = {Grassi, G. and Seravalle, G. and Quarti-Trevano, F. and Scopelliti, F. and Dell'Oro, R. and Bolla, G. and Mancia, G.},

title = {Excessive sympathetic activation in heart failure with obesity and metabolic syndrome: characteristics and mechanisms},

journal = {Hypertension},

volume = {49},

number = {3},

pages = {535-41},

ISSN = {1524-4563 (Electronic)

0194-911X (Linking)},

DOI = {10.1161/01.HYP.0000255983.32896.b9},

url = {<http://www.ncbi.nlm.nih.gov/pubmed/17210829>},

year = {2007},

type = {Journal Article}

}

@article{RN7,

author = {Groenland, E. H. and Spiering, W.},

title = {Baroreflex Amplification and Carotid Body Modulation for the Treatment of Resistant Hypertension},

journal = {Curr Hypertens Rep},

volume = {22},

number = {4},

pages = {27},

ISSN = {1534-3111 (Electronic)

1522-6417 (Linking)},

DOI = {10.1007/s11906-020-1024-x},

url = {<http://www.ncbi.nlm.nih.gov/pubmed/32166464>},

year = {2020},

type = {Journal Article}

}

@article{RN12,

author = {Guyton, A. C. and Coleman, T. G. and Granger, H. J.},

title = {Circulation: overall regulation},

journal = {Annu Rev Physiol},

volume = {34},

pages = {13-46},

ISSN = {0066-4278 (Print)

0066-4278 (Linking)},

DOI = {10.1146/annurev.ph.34.030172.000305},

url = {<http://www.ncbi.nlm.nih.gov/pubmed/4334846>},

year = {1972},

type = {Journal Article}

}

@article{RN24,

author = {Hall, J. E. and do Carmo, J. M. and da Silva, A. A. and Wang, Z. and Hall, M. E.},

title = {Obesity-induced hypertension: interaction of neurohumoral and renal mechanisms},

journal = {Circ Res},

volume = {116},

number = {6},

pages = {991-1006},

ISSN = {1524-4571 (Electronic)

0009-7330 (Linking)},

DOI = {10.1161/CIRCRESAHA.116.305697},

url = {<http://www.ncbi.nlm.nih.gov/pubmed/25767285>},

year = {2015},

type = {Journal Article}

}

@article{RN40,

author = {Hering, D. and Mahfoud, F. and Walton, A. S. and Krum, H. and Lambert, G. W. and Lambert, E. A. and Sobotka, P. A. and Bohm, M. and Cremers, B. and Esler, M. D. and Schlaich, M. P.},

title = {Renal denervation in moderate to severe CKD},

journal = {J Am Soc Nephrol},

volume = {23},

number = {7},

pages = {1250-7},

ISSN = {1533-3450 (Electronic)

1046-6673 (Linking)},

DOI = {10.1681/ASN.2011111062},

url = {<http://www.ncbi.nlm.nih.gov/pubmed/22595301>},

year = {2012},

type = {Journal Article}

}

@article{RN41,

author = {Hering, D. and Marusic, P. and Walton, A. S. and Lambert, E. A. and Krum, H. and Narkiewicz, K. and Lambert, G. W. and Esler, M. D. and Schlaich, M. P.},

title = {Sustained sympathetic and blood pressure reduction 1 year after renal denervation in patients with resistant hypertension},

journal = {Hypertension},

volume = {64},

number = {1},

pages = {118-24},

ISSN = {1524-4563 (Electronic)

0194-911X (Linking)},

DOI = {10.1161/HYPERTENSIONAHA.113.03098},

url = {<http://www.ncbi.nlm.nih.gov/pubmed/24732891>},

year = {2014},

type = {Journal Article}

}

@article{RN25,

author = {Hester, R. L. and Brown, A. J. and Husband, L. and Iliescu, R. and Pruett, D. and Summers, R. and Coleman, T. G.},

title = {HumMod: A Modeling Environment for the Simulation of Integrative Human Physiology},

journal = {Front Physiol},

volume = {2},

pages = {12},

ISSN = {1664-042X (Electronic)

1664-042X (Linking)},

DOI = {10.3389/fphys.2011.00012},

url = {<http://www.ncbi.nlm.nih.gov/pubmed/21647209>},

year = {2011},

type = {Journal Article}

}

@article{RN45,

author = {Hildebrandt, D. A. and Irwin, E. D. and Lohmeier, T. E.},

title = {Prolonged Baroreflex Activation Abolishes Salt-Induced Hypertension After Reductions in Kidney Mass},

journal = {Hypertension},

volume = {68},

number = {6},

pages = {1400-1406},

ISSN = {1524-4563 (Electronic)

0194-911X (Linking)},

DOI = {10.1161/HYPERTENSIONAHA.116.08293},

url = {<http://www.ncbi.nlm.nih.gov/pubmed/27777356>},

year = {2016},

type = {Journal Article}

}

@article{RN29,

author = {Hildebrandt, D. A. and Mizelle, H. L. and Brands, M. W. and Gaillard, C. A. and Smith, M. J., Jr. and Hall, J. E.},

title = {Intrarenal atrial natriuretic peptide infusion lowers arterial pressure chronically},

journal = {Am J Physiol},

volume = {259},

number = {3 Pt 2},

pages = {R585-92},

ISSN = {0002-9513 (Print)

0002-9513 (Linking)},

DOI = {10.1152/ajpregu.1990.259.3.R585},

url = {<http://www.ncbi.nlm.nih.gov/pubmed/2144407>},

year = {1990},

type = {Journal Article}

}

@article{RN51,

author = {Hoppe, U. C. and Brandt, M. C. and Wachter, R. and Beige, J. and Rump, L. C. and Kroon, A. A. and Cates, A. W. and Lovett, E. G. and Haller, H.},

title = {Minimally invasive system for baroreflex activation therapy chronically lowers blood pressure with pacemaker-like safety profile: results from the Barostim neo trial},

journal = {J Am Soc Hypertens},

volume = {6},

number = {4},

pages = {270-6},

ISSN = {1878-7436 (Electronic)

1878-7436 (Linking)},

DOI = {10.1016/j.jash.2012.04.004},

url = {<http://www.ncbi.nlm.nih.gov/pubmed/22694986>},

year = {2012},

type = {Journal Article}

}

@article{RN26,

author = {Huang, C. L. and Cogan, M. G.},

title = {Atrial natriuretic factor inhibits maximal tubuloglomerular feedback response},

journal = {Am J Physiol},

volume = {252},

number = {5 Pt 2},

pages = {F825-8},

ISSN = {0002-9513 (Print)

0002-9513 (Linking)},

DOI = {10.1152/ajprenal.1987.252.5.F825},

url = {<http://www.ncbi.nlm.nih.gov/pubmed/2953251>},

year = {1987},

type = {Journal Article}

}

@article{RN18,

author = {Iliescu, R. and Lohmeier, T. E.},

title = {Lowering of blood pressure during chronic suppression of central sympathetic outflow: insight from computer simulations},

journal = {Clin Exp Pharmacol Physiol},

volume = {37},

number = {2},

pages = {e24-33},

ISSN = {1440-1681 (Electronic)

0305-1870 (Linking)},

DOI = {10.1111/j.1440-1681.2009.05291.x},

url = {<http://www.ncbi.nlm.nih.gov/pubmed/19769610>},

year = {2010},

type = {Journal Article}

}

@article{RN30,

author = {Kurtz, A. and Della Bruna, R. and Pfeilschifter, J. and Taugner, R. and Bauer, C.},

title = {Atrial natriuretic peptide inhibits renin release from juxtaglomerular cells by a cGMP-mediated process},

journal = {Proc Natl Acad Sci U S A},

volume = {83},

number = {13},

pages = {4769-73},

ISSN = {0027-8424 (Print)

0027-8424 (Linking)},

url = {<http://www.ncbi.nlm.nih.gov/pubmed/3014509>},

year = {1986},

type = {Journal Article}

}

@article{RN23,

author = {Lohmeier, T. E. and Dwyer, T. M. and Irwin, E. D. and Rossing, M. A. and Kieval, R. S.},

title = {Prolonged activation of the baroreflex abolishes obesity-induced hypertension},

journal = {Hypertension},

volume = {49},

number = {6},

pages = {1307-14},

ISSN = {1524-4563 (Electronic)

0194-911X (Linking)},

DOI = {10.1161/HYPERTENSIONAHA.107.087874},

url = {<http://www.ncbi.nlm.nih.gov/pubmed/17438305>},

year = {2007},

type = {Journal Article}

}

@article{RN8,

author = {Lohmeier, T. E. and Hall, J. E.},

title = {Device-Based Neuromodulation for Resistant Hypertension Therapy},

journal = {Circ Res},

volume = {124},

number = {7},

pages = {1071-1093},

ISSN = {1524-4571 (Electronic)

0009-7330 (Linking)},

DOI = {10.1161/CIRCRESAHA.118.313221},

url = {<http://www.ncbi.nlm.nih.gov/pubmed/30920919>},

year = {2019},

type = {Journal Article}

}

@article{RN10,

author = {Lohmeier, T. E. and Hildebrandt, D. A. and Dwyer, T. M. and Barrett, A. M. and Irwin, E. D. and Rossing, M. A. and Kieval, R. S.},

title = {Renal denervation does not abolish sustained baroreflex-mediated reductions in arterial pressure},

journal = {Hypertension},

volume = {49},

number = {2},

pages = {373-9},

ISSN = {1524-4563 (Electronic)

0194-911X (Linking)},

DOI = {10.1161/01.HYP.0000253507.56499.bb},

url = {<http://www.ncbi.nlm.nih.gov/pubmed/17159083>},

year = {2007},

type = {Journal Article}

}

@article{RN6,

author = {Lohmeier, T. E. and Iliescu, R.},

title = {The baroreflex as a long-term controller of arterial pressure},

journal = {Physiology (Bethesda)},

volume = {30},

number = {2},

pages = {148-58},

ISSN = {1548-9221 (Electronic)

1548-9221 (Linking)},

DOI = {10.1152/physiol.00035.2014},

url = {<http://www.ncbi.nlm.nih.gov/pubmed/25729060>},

year = {2015},

type = {Journal Article}

}

@article{RN22,

author = {Lohmeier, T. E. and Iliescu, R. and Liu, B. and Henegar, J. R. and Maric-Bilkan, C. and Irwin, E. D.},

title = {Systemic and renal-specific sympathoinhibition in obesity hypertension},

journal = {Hypertension},

volume = {59},

number = {2},

pages = {331-8},

ISSN = {1524-4563 (Electronic)

0194-911X (Linking)},

DOI = {10.1161/HYPERTENSIONAHA.111.185074},

url = {<http://www.ncbi.nlm.nih.gov/pubmed/22184321>},

year = {2012},

type = {Journal Article}

}

@article{RN55,

author = {Lohmeier, T. E. and Iliescu, R. and Tudorancea, I. and Cazan, R. and Cates, A. W. and Georgakopoulos, D. and Irwin, E. D.},

title = {Chronic Interactions Between Carotid Baroreceptors and Chemoreceptors in Obesity Hypertension},

journal = {Hypertension},

volume = {68},

number = {1},

pages = {227-35},

ISSN = {1524-4563 (Electronic)

0194-911X (Linking)},

DOI = {10.1161/HYPERTENSIONAHA.116.07232},

url = {<http://www.ncbi.nlm.nih.gov/pubmed/27160198>},

year = {2016},

type = {Journal Article}

}

@article{RN5,

author = {McCubbin, J. W. and Green, J. H. and Page, I. H.},

title = {Baroceptor function in chronic renal hypertension},

journal = {Circ Res},

volume = {4},

number = {2},

pages = {205-10},

ISSN = {0009-7330 (Print)

0009-7330 (Linking)},

DOI = {10.1161/01.res.4.2.205},

url = {<http://www.ncbi.nlm.nih.gov/pubmed/13293821>},

year = {1956},

type = {Journal Article}

}

@article{RN28,

author = {Opgenorth, T. J. and Burnett, J. C., Jr. and Granger, J. P. and Scriven, T. A.},

title = {Effects of atrial natriuretic peptide on renin secretion in nonfiltering kidney},

journal = {Am J Physiol},

volume = {250},

number = {5 Pt 2},

pages = {F798-801},

ISSN = {0002-9513 (Print)

0002-9513 (Linking)},

DOI = {10.1152/ajprenal.1986.250.5.F798},

url = {<http://www.ncbi.nlm.nih.gov/pubmed/2939730>},

year = {1986},

type = {Journal Article}

}

@article{RN31,

author = {Pollock, D. M. and Arendshorst, W. J.},

title = {Native tubular fluid attenuates ANF-induced inhibition of tubuloglomerular feedback},

journal = {Am J Physiol},

volume = {258},

number = {1 Pt 2},

pages = {F189-98},

ISSN = {0002-9513 (Print)

0002-9513 (Linking)},

DOI = {10.1152/ajprenal.1990.258.1.F189},

url = {<http://www.ncbi.nlm.nih.gov/pubmed/2137295>},

year = {1990},

type = {Journal Article}

}

@article{RN20,

author = {Pruett, W. A. and Clemmer, J. S. and Hester, R. L.},

title = {Validation of an Integrative Mathematical Model of Dehydration and Rehydration in Virtual Humans},

journal = {Physiol Rep},

volume = {4},

number = {22},

pages = {22},

ISSN = {1600-6143 (Electronic)

1600-6135 (Linking)},

DOI = {10.1111/ajt.14028},

url = {<http://www.ncbi.nlm.nih.gov/pubmed/27696661>},

year = {2016},

type = {Journal Article}

}

@article{RN19,

author = {Pruett, W. A. and Husband, L. D. and Husband, G. and Dakhlalla, M. and Bellamy, K. and Coleman, T. G. and Hester, R. L.},

title = {A population model of integrative cardiovascular physiology},

journal = {PLoS One},

volume = {8},

number = {9},

pages = {e74329},

ISSN = {1932-6203 (Electronic)

1932-6203 (Linking)},

DOI = {10.1371/journal.pone.0074329},

url = {<http://www.ncbi.nlm.nih.gov/pubmed/24058546>},

year = {2013},

type = {Journal Article}

}

@article{RN44,

author = {Reisin, E. and Weir, M. R. and Falkner, B. and Hutchinson, H. G. and Anzalone, D. A. and Tuck, M. L.},

title = {Lisinopril versus hydrochlorothiazide in obese hypertensive patients: a multicenter placebo-controlled trial. Treatment in Obese Patients With Hypertension (TROPHY) Study Group},

journal = {Hypertension},

volume = {30},

number = {1 Pt 1},

pages = {140-5},

ISSN = {0194-911X (Print)

0194-911X (Linking)},

DOI = {10.1161/01.hyp.30.1.140},

url = {<http://www.ncbi.nlm.nih.gov/pubmed/9231834>},

year = {1997},

type = {Journal Article}

}

@article{RN53,

author = {Richards, M. and Espiner, E. and Frampton, C. and Ikram, H. and Yandle, T. and Sopwith, M. and Cussans, N.},

title = {Inhibition of endopeptidase EC 24.11 in humans. Renal and endocrine effects},

journal = {Hypertension},

volume = {16},

number = {3},

pages = {269-76},

ISSN = {0194-911X (Print)

0194-911X (Linking)},

DOI = {10.1161/01.hyp.16.3.269},

url = {<http://www.ncbi.nlm.nih.gov/pubmed/2144260>},

year = {1990},

type = {Journal Article}

}

@article{RN54,

author = {Ruilope, L. M. and Dukat, A. and Bohm, M. and Lacourciere, Y. and Gong, J. and Lefkowitz, M. P.},

title = {Blood-pressure reduction with LCZ696, a novel dual-acting inhibitor of the angiotensin II receptor and neprilysin: a randomised, double-blind, placebo-controlled, active comparator study},

journal = {Lancet},

volume = {375},

number = {9722},

pages = {1255-66},

ISSN = {1474-547X (Electronic)

0140-6736 (Linking)},

DOI = {10.1016/S0140-6736(09)61966-8},

url = {<http://www.ncbi.nlm.nih.gov/pubmed/20236700>},

year = {2010},

type = {Journal Article}

}

@article{RN35,

author = {Rumantir, M. S. and Vaz, M. and Jennings, G. L. and Collier, G. and Kaye, D. M. and Seals, D. R. and Wiesner, G. H. and Brunner-La Rocca, H. P. and Esler, M. D.},

title = {Neural mechanisms in human obesity-related hypertension},

journal = {J Hypertens},

volume = {17},

number = {8},

pages = {1125-33},

ISSN = {0263-6352 (Print)

0263-6352 (Linking)},

DOI = {10.1097/00004872-199917080-00012},

url = {<http://www.ncbi.nlm.nih.gov/pubmed/10466468>},

year = {1999},

type = {Journal Article}

}

@article{RN32,

author = {Salazar, F. J. and Fiksen-Olsen, M. J. and Opgenorth, T. J. and Granger, J. P. and Burnett, J. C., Jr. and Romero, J. C.},

title = {Renal effects of ANP without changes in glomerular filtration rate and blood pressure},

journal = {Am J Physiol},

volume = {251},

number = {3 Pt 2},

pages = {F532-6},

ISSN = {0002-9513 (Print)

0002-9513 (Linking)},

DOI = {10.1152/ajprenal.1986.251.3.F532},

url = {<http://www.ncbi.nlm.nih.gov/pubmed/2944393>},

year = {1986},

type = {Journal Article}

}

@article{RN13,

author = {Summers, R. L. and Coleman, T. G.},

title = {Computer systems analysis of the cardiovascular mechanisms of reentry orthostasis in astronauts},

journal = {Comput Cardiol},

volume = {29},

pages = {521-4},

ISSN = {0276-6574 (Print)

0276-6574 (Linking)},

url = {<http://www.ncbi.nlm.nih.gov/pubmed/14686452>},

year = {2002},

type = {Journal Article}

}

@article{RN14,

author = {Summers, R. L. and Harrison, J. M. and Thompson, J. R. and Porter, J. and Coleman, T. G.},

title = {Theoretical analysis of the effect of positioning on hemodynamic stability during pregnancy},

journal = {Acad Emerg Med},

volume = {18},

number = {10},

pages = {1094-8},

ISSN = {1553-2712 (Electronic)

1069-6563 (Linking)},

DOI = {10.1111/j.1553-2712.2011.01166.x},

url = {<http://www.ncbi.nlm.nih.gov/pubmed/21951760>},

year = {2011},

type = {Journal Article}

}

@article{RN15,

author = {Summers, R. L. and Martin, D. S. and Meck, J. V. and Coleman, T. G.},

title = {Computer systems analysis of spaceflight induced changes in left ventricular mass},

journal = {Comput Biol Med},

volume = {37},

number = {3},

pages = {358-63},

ISSN = {0010-4825 (Print)

0010-4825 (Linking)},

DOI = {10.1016/j.compbiomed.2006.04.003},

url = {<http://www.ncbi.nlm.nih.gov/pubmed/16808910>},

year = {2007},

type = {Journal Article}

}

@article{RN16,

author = {Summers, R. L. and Ward, K. R. and Witten, T. and Convertino, V. A. and Ryan, K. L. and Coleman, T. G. and Hester, R. L.},

title = {Validation of a computational platform for the analysis of the physiologic mechanisms of a human experimental model of hemorrhage},

journal = {Resuscitation},

volume = {80},

number = {12},

pages = {1405-10},

ISSN = {1873-1570 (Electronic)

0300-9572 (Linking)},

DOI = {10.1016/j.resuscitation.2009.09.001},

url = {<http://www.ncbi.nlm.nih.gov/pubmed/19804937>},

year = {2009},

type = {Journal Article}

}

@article{RN36,

author = {Vaz, M. and Jennings, G. and Turner, A. and Cox, H. and Lambert, G. and Esler, M.},

title = {Regional sympathetic nervous activity and oxygen consumption in obese normotensive human subjects},

journal = {Circulation},

volume = {96},

number = {10},

pages = {3423-9},

ISSN = {0009-7322 (Print)

0009-7322 (Linking)},

DOI = {10.1161/01.cir.96.10.3423},

url = {<http://www.ncbi.nlm.nih.gov/pubmed/9396437>},

year = {1997},

type = {Journal Article}

}

@article{RN27,

author = {Villarreal, D. and Freeman, R. H. and Davis, J. O. and Verburg, K. M. and Vari, R. C.},

title = {Renal mechanisms for suppression of renin secretion by atrial natriuretic factor},

journal = {Hypertension},

volume = {8},

number = {6 Pt 2},

pages = {II28-35},

ISSN = {0194-911X (Print)

0194-911X (Linking)},

url = {<http://www.ncbi.nlm.nih.gov/pubmed/2941371>},

year = {1986},

type = {Journal Article}

}

@article{RN50,

author = {Wallbach, M. and Halbach, M. and Reuter, H. and Passauer, J. and Luders, S. and Bohning, E. and Zenker, D. and Muller, G. A. and Wachter, R. and Koziolek, M. J.},

title = {Baroreflex activation therapy in patients with prior renal denervation},

journal = {J Hypertens},

volume = {34},

number = {8},

pages = {1630-8},

ISSN = {1473-5598 (Electronic)

0263-6352 (Linking)},

DOI = {10.1097/HJH.0000000000000949},

url = {<http://www.ncbi.nlm.nih.gov/pubmed/27137174>},

year = {2016},

type = {Journal Article}

}

@article{RN42,

author = {Wallbach, M. and Lehnig, L. Y. and Schroer, C. and Luders, S. and Bohning, E. and Muller, G. A. and Wachter, R. and Koziolek, M. J.},

title = {Effects of Baroreflex Activation Therapy on Ambulatory Blood Pressure in Patients With Resistant Hypertension},

journal = {Hypertension},

volume = {67},

number = {4},

pages = {701-9},

ISSN = {1524-4563 (Electronic)

0194-911X (Linking)},

DOI = {10.1161/HYPERTENSIONAHA.115.06717},

url = {<http://www.ncbi.nlm.nih.gov/pubmed/26902491>},

year = {2016},

type = {Journal Article}

}
