# Supplementary material for: In silico trial of baroreflex activation therapy for the treatment of obesity-induced hypertension
Source: PLoS One. 2021 Nov 18;16(11):e0259917. doi: 10.1371/journal.pone.0259917 (PMC8601446; doi:10.1371/journal.pone.0259917)
Supplement: S1 Fig — These factors included atrial natriuretic peptide (ANP), renal sympathetic nerve activity (RSNA), angiotensin II (ANG II), norepinephrine (NE), and epinephrine (Epi). *p<0.05; #p<0.05 vs. baseline. (PDF) [file pone.0259917.s002.pdf]

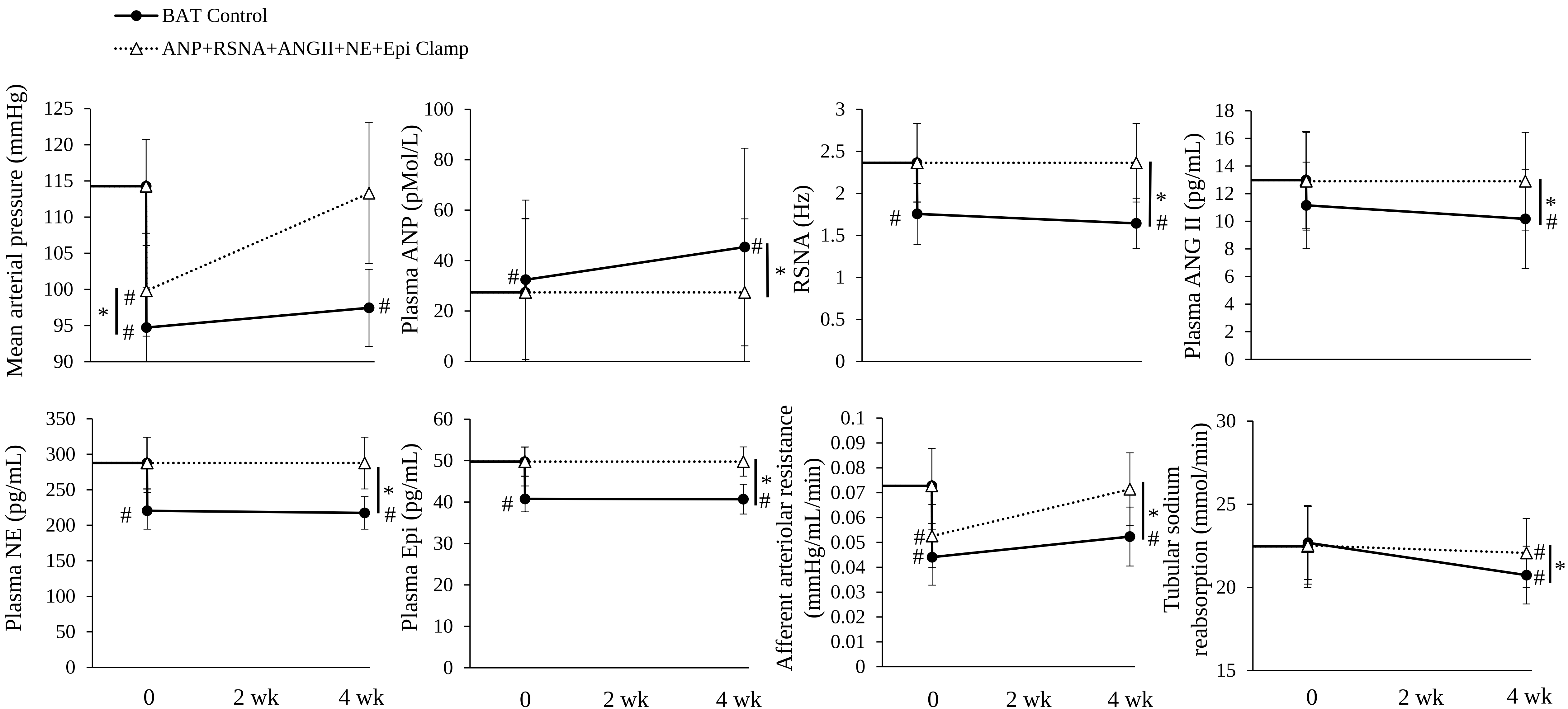

Supplementary Figure 1. Cardiovascular and renal responses to BAT in a control simulation (BAT Control) and when several factors were clamped at baseline. These factors included atrial natriuretic peptide (ANP), renal sympathetic nerve activity (RSNA), angiotensin II (ANG II), norepinephrine (NE), and epinephrine (Epi). *p<0.05; #p<0.05 vs. baseline
