# Supplementary material for: In silico trial of baroreflex activation therapy for the treatment of obesity-induced hypertension
Source: PLoS One. 2021 Nov 18;16(11):e0259917. doi: 10.1371/journal.pone.0259917 (PMC8601446; doi:10.1371/journal.pone.0259917)
Supplement: S2 Fig — FF indicates filtration fraction; SNGFR, single nephron glomerular filtration rate; PTconductance, conductance of the proximal tubule; RBF, renal blood flow; Kf, filtration coefficient; PC, capillary hydrostatic pressure; PBC, Bowman’s Capsule hydrostatic pressure; Posm, capillary colloid osmotic pressure; and RPF, renal plasma flow. *Indicates implicit equation. (PDF) [file pone.0259917.s003.pdf]

Supplementary Figure 2. Equations and model parameters for calculating glomerular filtration rate

**Variable**	**Input**
Colloid Osmotic Pressure =	Plasma Osmotic Pressure / (1 - FF)
Bowman's Capsule Pressure =	SNGFR / PTconductance
Capillary Hydrostatic Pressure =	(RBF / Efferent Conductance) + Renal Venous Pressure

$$\mathbf{GFR = Kf\ (P_{C} - P_{BC} - P_{osm})}$$

*FF = GFR / RPF

*SNGFR = GFR / Nephron #

PTconductance = 2.55 x 10^{-6}

Kf = 8.89 x Nephron # (xNormal)

FF indicates filtration fraction; SNGFR, single nephron glomerular filtration rate; PTconductance, conductance of the proximal tubule; RBF, renal blood flow; Kf, filtration coefficient; P_C, capillary hydrostatic pressure; P_{BC}, Bowman's Capsule hydrostatic pressure; P_{osm}, capillary colloid osmostic pressu
and RPF, renal plasma flow.

*Indicates implicit equation
