# Supplementary material for: In silico trial of baroreflex activation therapy for the treatment of obesity-induced hypertension
Source: PLoS One. 2021 Nov 18;16(11):e0259917. doi: 10.1371/journal.pone.0259917 (PMC8601446; doi:10.1371/journal.pone.0259917)
Supplement: S3 Fig — Ang II indicates angiotensin II; SM, skeletal muscle; PO2, partial pressure of oxygen; ADH, antidiuretic hormone; GI, gastrointestinal; PCO2, partial pressure of carbon dioxide; and temp, temperature. *Indicates a negative relationship. (PDF) [file pone.0259917.s004.pdf]

Supplementary Figure 3. Peripheral blood flow and determinants of organ conductances in the model

Organ	Effect	Effect Range	Input
Skeletal Muscle Conductance	**Ang II Effect***	0.5 - 1.05	[Ang II]
	**Sympathetic Effect***	0.3 - 1.3	SM α_1 Receptor Activation
	**Sympathetic Effect**	0.5 - 1.75	SM β_2 Receptor Activation
	**Delayed PO_2 Effect***	0 - 1.2	SM PO_2
	**Acute PO_2 Effect***	0.4 - 6	SM PO_2
	**ADH Effect***	0.1 - 1	[ADH]
	**Metabolism Effect**	1.0 - 3	Metabolic Oxygen Need
	**Muscle Pump Effect**	1.0 - 3	Intensity and Rate of Exercise
	**CCB Effect**	1.0 - 4	[Isradipine]
Baseline conductance (ml/min/mmHg/g) = 0.00029			
GI Tract Conductance	**Ang II Effect***	0.5 - 1.05	[Ang II]
	**Sympathetic Effect***	0.1 - 1.3	GI α_1 Receptor Activation
	**Delayed PO_2 Effect***	0.2 - 1.2	GI PO_2
	**Acute PO_2 Effect***	0.2 - 2	GI PO_2
	**ADH Effect**	0.1 - 1	[ADH]
	**CCB Effect**	1.0 - 4	[Isradipine]
Baseline conductance (ml/min/mmHg/g) = 0.00904			
Fat Conductance	**Ang II Effect***	0.5 - 1.05	[Ang II]
	**Sympathetic Effect***	0.1 - 1.3	Fat α_1 Receptor Activation
	**ADH Effect***	0.1 - 1	[ADH]
	**Delayed PO_2 Effect***	0.8 - 1.2	Fat PO_2
	**Acute PO_2 Effect***	0.4 - 2	Fat PO_2
	**CCB Effect**	1.0 - 4	[Isradipine]
Baseline conductance (ml/min/mmHg/g) = 0.00019			
Bone Conductance	**Ang II Effect***	0.5 - 1.05	[Ang II]
	**Sympathetic Effect***	0.1 - 1.3	Bone α_1 Receptor Activation
	**ADH Effect***	0.1 - 1	[ADH]
	**Delayed PO_2 Effect***	0.8 - 1.2	Bone PO_2
	**Acute PO_2 Effect***	0.4 - 2	Bone PO_2
Baseline conductance (ml/min/mmHg/g) = 0.00029			

Ang II indicates angiotensin II; SM, skeletal muscle; PO_2, partial pressure of oxygen; ADH, antidiuretic hormone; GI, gastrointestinal; PCO_2 , partial pressure of carbon dioxide; and temp, temperature.

*Indicates a negative relationship

Supplementary Figure 3 (continued). Peripheral blood flow and determinants of organ conductances

Organ	Effect	Effect Range	Input
Brain Conductance	**Delayed PO_2 Effect***	0.8 - 1.2	Brain PO_2
	**Acute PO_2 Effect***	0.9 - 2.2	Brain PO_2
	**Acute PCO_2 Effect**	0.7- 2.2	Brain PCO_2
Baseline conductance (ml/min/mmHg/g) = 0.00597			
Skin Conductance	**Ang II Effect***	0.5 - 1.05	[Ang II]
	**Sympathetic Effect***	0.1 - 1.3	Other Tissue α_1 Receptor Activation
	**Body Temp Effect**	0.3 - 8	(Core Temp - 37 °C)
	**Local Temp Effect**	0.2 - 5	Skin Temp
	**ADH Effect***	0.1 - 1	[ADH]
	**Delayed PO_2 Effect***	0.8 - 1.2	Skin PO_2
	**Acute PO_2 Effect***	0.2 - 2	Skin PO_2
Baseline conductance (ml/min/mmHg/g) = 0.00112			
Liver Conductance	**Sympathetic Effect***	0.1 - 1.3	Hepatic Artery α_1 Receptor Activation
Baseline conductance (ml/min/mmHg/g) = 0.00187			
Other Tissue Conductance	**Ang II Effect***	0.5 - 1.05	[Ang II]
	**Sympathetic Effect***	0.1 - 1.3	Tissue α_1 Receptor Activation
	**ADH Effect***	0.1 - 1	[ADH]
	**Delayed PO_2 Effect***	0.8 - 1.2	Tissue PO_2
	**Acute PO_2 Effect***	0.2 - 2	Tissue PO_2
	**CCB Effect**	1.0 - 4	[Isradipine]
Baseline conductance (ml/min/mmHg/g) = 0.00141			

Ang II indicates angiotensin II; SM, skeletal muscle; PO_2, partial pressure of oxygen; ADH, antidiuretic hormone; GI, gastrointestinal; PCO_2 , partial pressure of carbon dioxide; and temp, temperature.

*Indicates a negative relationship
