# Supplementary material for: In silico trial of baroreflex activation therapy for the treatment of obesity-induced hypertension
Source: PLoS One. 2021 Nov 18;16(11):e0259917. doi: 10.1371/journal.pone.0259917 (PMC8601446; doi:10.1371/journal.pone.0259917)
Supplement: S4 Fig — Symp indicates sympathetic; ANP, atrial natriuretic peptide; myo, myogenic; TGF, tubuloglomerular feedback; CCB, calcium channel blocker; and Ang II, angiotensin II. Data point on each relationship indicates normal model values. *Myogenic effect resets within 12 hours. (PDF) [file pone.0259917.s005.pdf]

Supplementary Figure 4. Determinants of afferent and efferent arteriolar conductance in the model

Afferent Arteriolar Conductance

$$Conductance = Baseline\ \times Symp\ Effect\ \times ANP\ Effect\ \times Myogenic\ Effect\ \times TGF\ Effect\ \times CCB\ Effect$$

$$Baseline = 0.080883\quad ml/min/mmHg/g$$

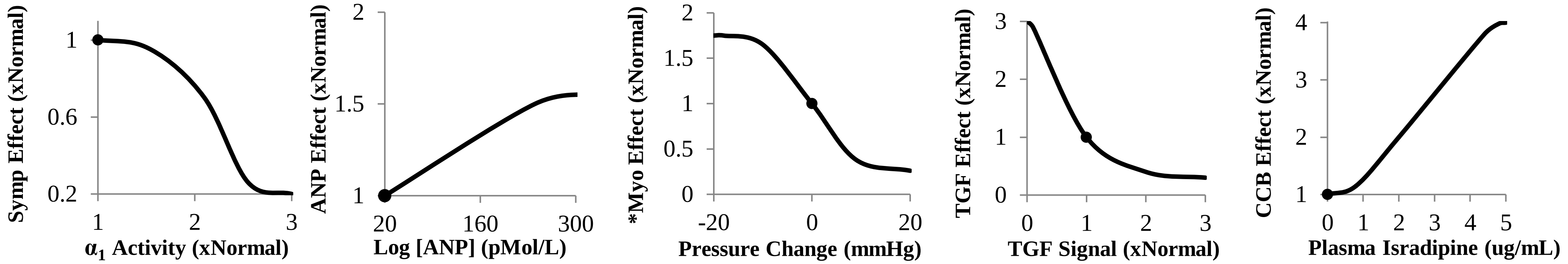

Efferent Arteriolar Conductance

$$Conductance = Baseline\ \times Symp\ Effect\ \times Ang\ II\ Effect\ \times LT\ CCB\ Effect$$

$$Baseline = 0.06218\quad ml/min/mmHg/g$$

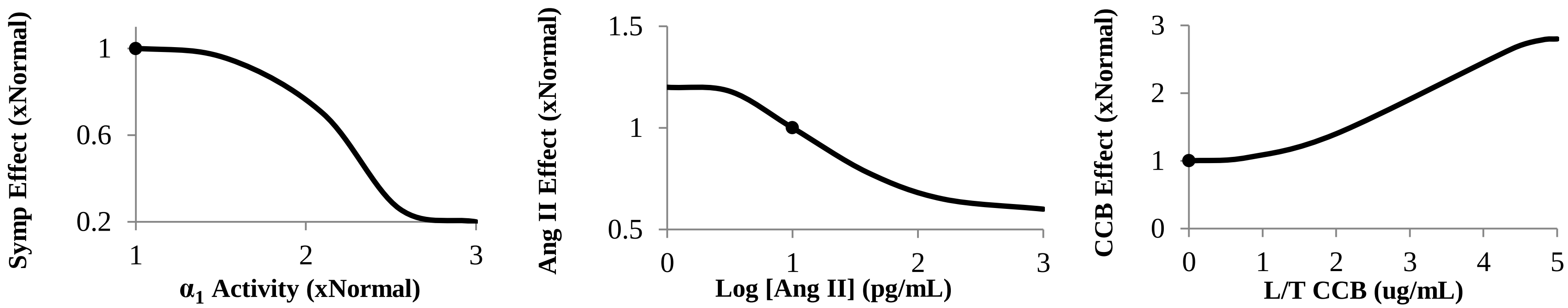

Symp indicates sympathetic; ANP, atrial natriuretic peptide; myo, myogenic; TGF, tubuloglomerular feedback; CCB, calcium channel blocker; and Ang II, angiotensin II.

Data point on each relationship indicates normal model values.

*Myogenic effect resets within 12 hours.
