# Supplementary material for: In silico trial of baroreflex activation therapy for the treatment of obesity-induced hypertension
Source: PLoS One. 2021 Nov 18;16(11):e0259917. doi: 10.1371/journal.pone.0259917 (PMC8601446; doi:10.1371/journal.pone.0259917)
Supplement: S5 Fig — (PDF) [file pone.0259917.s006.pdf]

Supplementary Figure 5. Determinants of tubuloglomerular feedback

TGF Signal

$$Signal = \boldsymbol{Sodium\ Effect} \times \boldsymbol{Ang\ II\ Effect} \times \boldsymbol{ANP\ Effect}$$

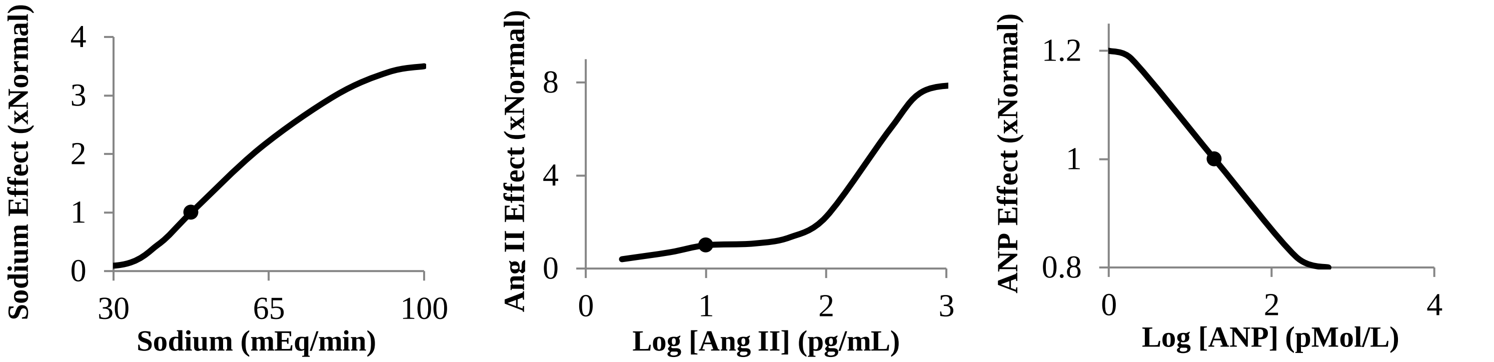

Ang II indicates angiotensin II; and ANP, atrial natriuretic peptide.
