# Supplementary material for: In silico trial of baroreflex activation therapy for the treatment of obesity-induced hypertension
Source: PLoS One. 2021 Nov 18;16(11):e0259917. doi: 10.1371/journal.pone.0259917 (PMC8601446; doi:10.1371/journal.pone.0259917)
Supplement: S6 Fig — MD indicates macula densa; symp, sympathetic; and ANP, atrial natriuretic peptide. (PDF) [file pone.0259917.s007.pdf]

Supplementary Figure 6. Determinants of renin secretion in the model

Renin Secretion

$$Renin\ Synthesis = \boldsymbol{MD\ Effect} \times \boldsymbol{Symp\ Effect} \times \boldsymbol{ANPEffect} \times Renal\ Mass\ \% \times Base$$

$$Renin\ Mass = Renin\ Synthesis - Renin\ Secretion$$

$$Renin\ Secretion = \boldsymbol{MD\ Effect} \times \boldsymbol{Symp\ Effect} \times \boldsymbol{ANPEffect} \times Renin\ Mass \times K$$

$Base\ synthesis = 160\ GU/min$

$K = 0.00165$

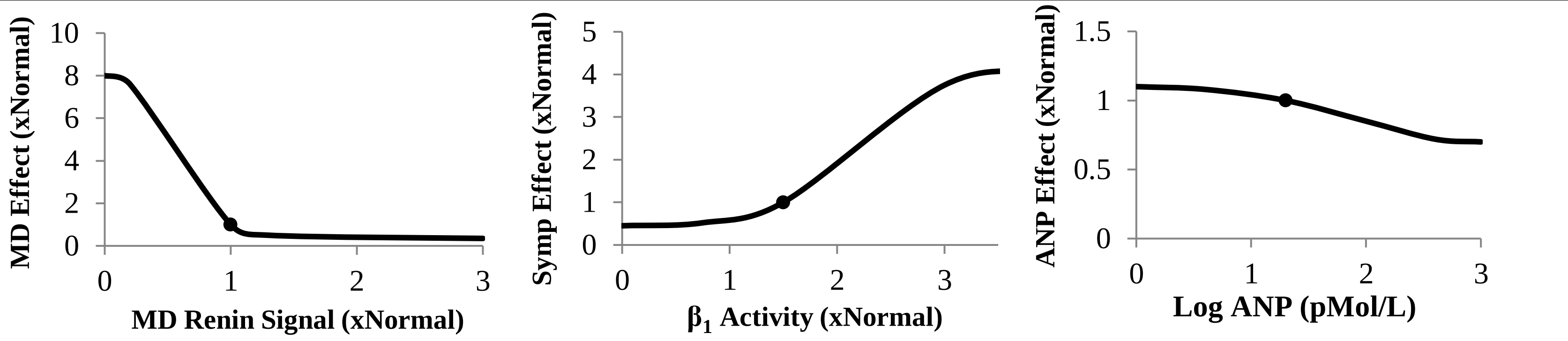

MD indicates macula densa; symp, sympathetic; and ANP, atrial natriuretic peptide.
