# Supplementary figures and images for: In silico trial of baroreflex activation therapy for the treatment of obesity-induced hypertension
Source: PLoS One. 2021 Nov 18;16(11):e0259917. doi: 10.1371/journal.pone.0259917 (PMC8601446; doi:10.1371/journal.pone.0259917)

Supplementary Figure 7. Body compartment volumes

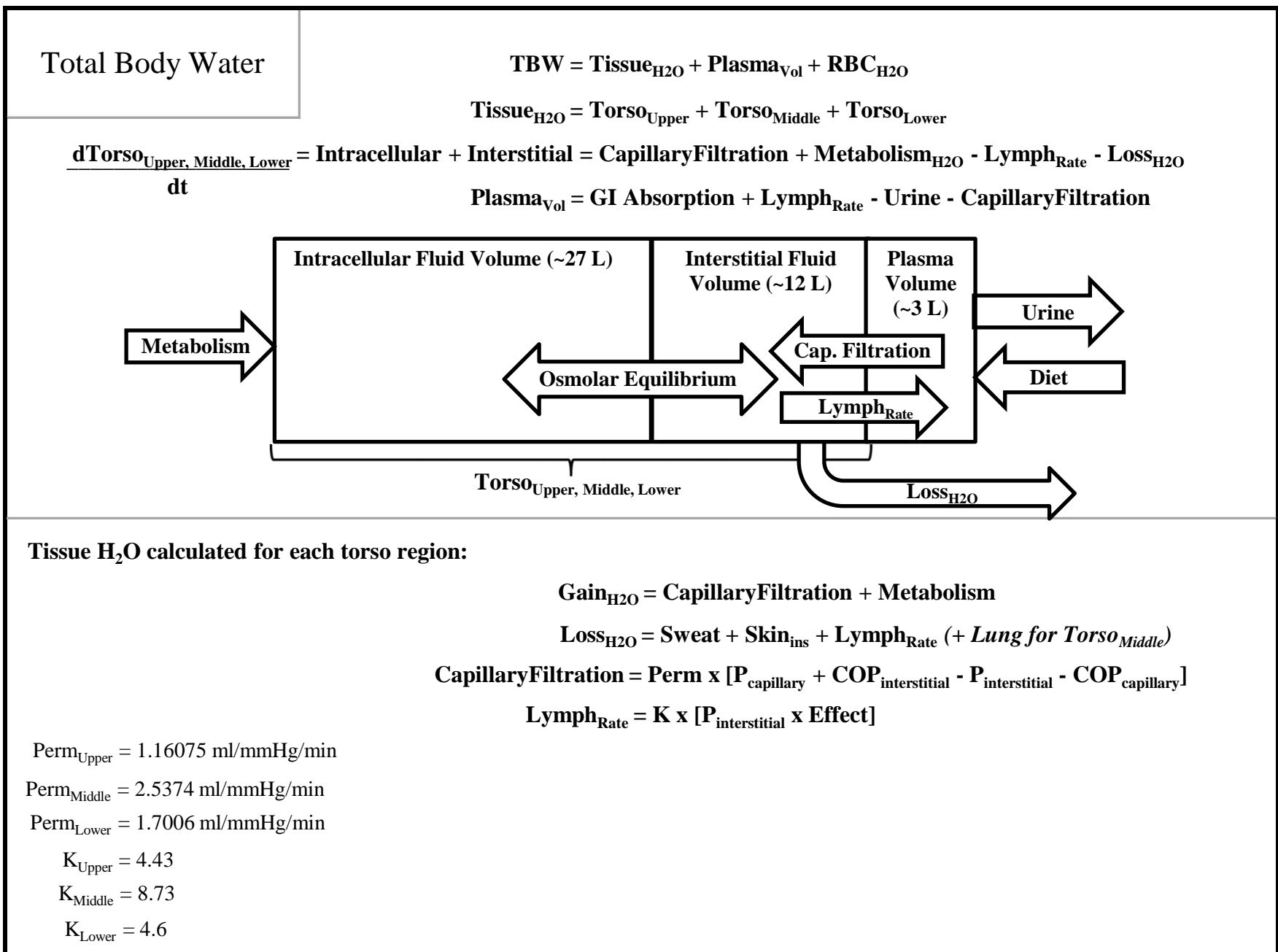

Supplement: S7 Fig — (PDF) [file pone.0259917.s008.pdf]
