# Supplementary material for: In silico trial of baroreflex activation therapy for the treatment of obesity-induced hypertension
Source: PLoS One. 2021 Nov 18;16(11):e0259917. doi: 10.1371/journal.pone.0259917 (PMC8601446; doi:10.1371/journal.pone.0259917)
Supplement: S8 Fig — PTNa indicates proximal tubular sodium; angiotensin II, Ang II; sympathetic, symp; atrial natriuretic peptide, ANP; and renal interstitial fluid pressure, RIFP. (PDF) [file pone.0259917.s009.pdf]

Supplementary Figure 8. Determinants of proximal tubular sodium reabsorption

Proximal Tubule Sodium Reabsorption

$$Reabsorption = PTNa\ Inflow\ \times Baseline^{\ Effects^{-1}}$$

$$Baseline = 58\%$$

$$Effects = \boldsymbol{Ang\ II\ Effect}\ \times \boldsymbol{Symp\ Effect}\ \times \boldsymbol{ANP\ Effect}\ \times \boldsymbol{RIFP\ Effect}$$

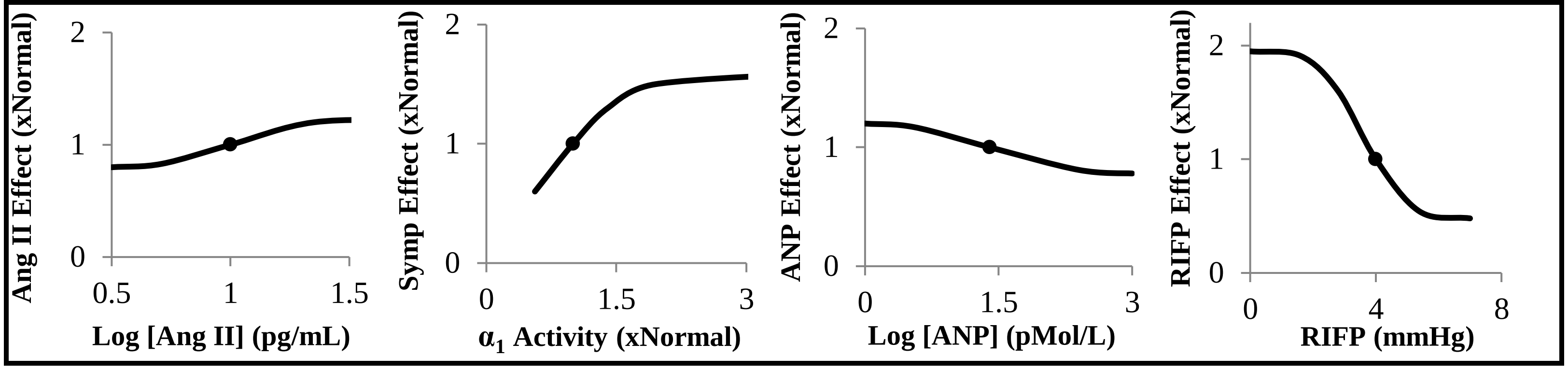

PTNa indicates proximal tubular sodium; angiotensin II, Ang II; sympathetic, symp; atrial natriuretic peptide, ANP; renal interstitial fluid pressure, RIFP
