# Supplementary material for: In silico trial of baroreflex activation therapy for the treatment of obesity-induced hypertension
Source: PLoS One. 2021 Nov 18;16(11):e0259917. doi: 10.1371/journal.pone.0259917 (PMC8601446; doi:10.1371/journal.pone.0259917)
Supplement: S11 Fig — Single nephron, SN; aldosterone, Aldo. (PDF) [file pone.0259917.s012.pdf]

Supplementary Figure 11. Determinants of collecting duct sodium reabsorption

Collecting Duct Sodium Reabsorption

$$Reabsorption = Loop\ Inflow \times Baseline^{\ Effects^{-1}}$$

$$Baseline = 75\%$$

$$Effects = \boldsymbol{Sodium\ Effect} \times \boldsymbol{ANP\ Effect}$$

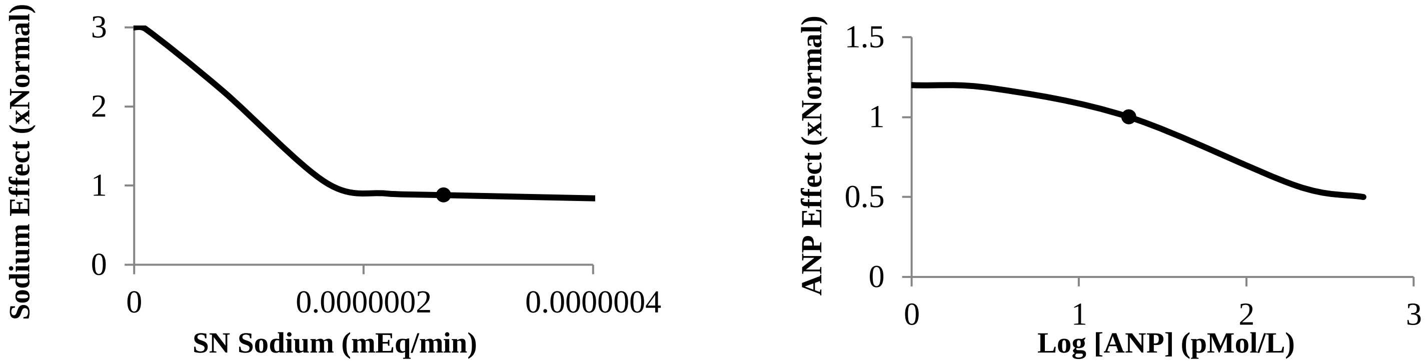

Single nephron, SN; aldosterone, Aldo
